# Multi-trait genome prediction of new environments with partial least squares

**DOI:** 10.3389/fgene.2022.966775

**Published:** 2022-09-05

**Authors:** Osval A. Montesinos-López, Abelardo Montesinos-López, David Alejandro Bernal Sandoval, Brandon Alejandro Mosqueda-Gonzalez, Marco Alberto Valenzo-Jiménez, José Crossa

**Affiliations:** ^1^ Facultad de Telemática, Universidad de Colima, Colima, Mexico; ^2^ Centro Universitario de Ciencias Exactas e Ingenierías (CUCEI), Universidad de Guadalajara, Guadalajara, Mexico; ^3^ Centro de Investigación en Computación (CIC), Instituto Politécnico Nacional (IPN), Mexico City, Mexico; ^4^ Universidad Michoacana de San Nicolas de Hidalgo (UMSNH), Avenida Francisco J. Mujica S/N Ciudad Universitaria, Morelia, MC, Mexico; ^5^ International Maize and Wheat Improvement Center, Texcoco, Edo. de Mexico, Mexico; ^6^ Colegio de Porstgraduados, Montecillos, Edo. de Mexico, Mexico

**Keywords:** genomic prediction, genotype × environment interaction, multi-trait partial least squares, single-trait partial least squares, prediction of one complete environment

## Abstract

The genomic selection (GS) methodology proposed over 20 years ago by Meuwissen et al. (Genetics, 2001) has revolutionized plant breeding. A predictive methodology that trains statistical machine learning algorithms with phenotypic and genotypic data of a reference population and makes predictions for genotyped candidate lines, GS saves significant resources in the selection of candidate individuals. However, its practical implementation is still challenging when the plant breeder is interested in the prediction of future seasons or new locations and/or environments, which is called the “leave one environment out” issue. Furthermore, because the distributions of the training and testing set do not match, most statistical machine learning methods struggle to produce moderate or reasonable prediction accuracies. For this reason, the main objective of this study was to explore the use of the multi-trait partial least square (MT-PLS) regression methodology for this specific task, benchmarking its performance with the Bayesian Multi-trait Genomic Best Linear Unbiased Predictor (MT-GBLUP) method. The benchmarking process was performed with five actual data sets. We found that in all data sets the MT-PLS method outperformed the popular MT-GBLUP method by 349.8% (under predictor E + G), 484.4% (under predictor E + G + GE; where E denotes environments, G genotypes and GE the genotype by environment interaction) and 15.9% (under predictor G + GE) across traits. Our results provide empirical evidence of the power of the MT-PLS methodology for the prediction of future seasons or new environments. Furthermore, the comparison between single univariate-trait (UT) versus MT for GBLUP and PLS gave an increase in prediction accuracy of MT-GBLUP versus UT-GBLUP, but not for MT-PLS versus UT-PLS.

## Introduction

In genomic assisted plant breeding, improving the prediction of future years or new locations and/or environments (leave one environment out), is necessary to increase the genetic gain in breeding programs. Genomic selection (GS) has the potential to increase the prediction accuracy of future seasons or new locations because it is based on a predictive methodology. Multi-trait (MT) models are key tools for improving prediction accuracy in genomic selection (GS). For example, MT models offer benefits over single-trait (unit-trait, UT) models when the traits under study are correlated, and in addition, allow the computation of an optimal and simplified total merit selection index (Okeke et al., 2017). Most existing models for genomic prediction are UT models, and few are used for MT genomic prediction even though MT models offer many advantages. UT models are trained to predict a single trait at a time (continuous, binary, categorical or count), while MT models are trained to simultaneously predict at least two traits.

MT models are preferred over UT models because: 1) they more efficiently represent complex relationships between traits, 2) simultaneously exploit the correlation between lines and traits, 3) offer better interpretability than UT models, 4) are more efficient to train computationally than each UT model separately, 5) improve index selection since more precise estimates of random effects of lines and genetic correlation between traits are obtained, 6) improve indirect selection because of increased precision of genetic correlation parameter estimates between traits, and 7) improve hypotheses to reduce type I and II errors. A type I error (false-positive) occurs when the investigator rejects a null hypothesis that is true; a type II error (false-negative) occurs when the investigator fails to reject a null hypothesis that is false (Montesinos-López et al., 2019a).

There is empirical evidence that MT models can increase prediction accuracy of low heritability traits that have at least moderate correlation with high heritability traits (Jia and Jannink, 2012; Montesinos-López et al., 2016). In general, when the traits are at least moderately correlated, MT models improve parameter estimates and prediction accuracy as compared to UT models as reported by Schulthess et al. (2017). Calus and Veerkamp (2011), Jia and Jannink (2012), Jiang et al. (2015), Montesinos-López et al. (2016), He et al. (2016) and Schulthess et al. (2017) reported better prediction accuracies of MT models with respect to UT models. These authors have also documented the efficiency of MT models for predicting expensive traits that are correlated with inexpensive secondary traits, as MT models are helpful in developing better genomic selection strategies. Montesinos-López et al. (2019a) proposed an improved Bayesian multi-trait and multi-environment (BMTME) R package that implements the BMTME model (Montesinos-López et al., 2016) and is able to capture the correlation not only between lines, but also between traits and environments. Additionally, this package allows the Bayesian multi-output regressor stacking (BMORS) functions to be implemented, which are considerably efficient in terms of computational resources.

In the statistical literature, MT models are known as multivariate models and have been implemented in many areas, including environmental science, education, chemistry, telecommunications, psychology, medicine, communications, engineering, and food science. MT models are better than UT models because of improved parameter estimates and prediction accuracy. With the continuous and dramatic growth of computational power, MT models play an increasingly important role in data analysis in plant and animal genomic-assisted breeding for selecting the best candidate genotypes.

However, the use of MT models is not as widespread as UT models because: 1) there is a lack of friendly software for performing MT analyses; 2) there are insufficient computational resources since fitting MT models is more demanding than fitting UT models; 3) MT models have more complex genotype × environment interactions (GE) since traits have different response patterns in different environments; 4) it is more difficult to assess and achieve MT model assumptions; 5) MT models have more problems of convergence than UT models; and 6) implementing MT models for genomic prediction is more challenging due to the size and complexity of the underlying data sets (Montesinos-López et al., 2019b).

With the goal of utilizing MT models for genomic prediction, some models have been proposed for GS; the two most popular methods are multi-trait mixed models and their Bayesian version, Bayesian Multi-Trait Genomic Best Linear Unbiased Predictor (MT-GBLUP). Multi-trait models under artificial deep neural networks have even been explored in genomic selection (Montesinos-López et al., 2018; 2019c). Recently, Montesinos-López et al. (2022a) explored the use of the partial least square (PLS) regression methodology for the prediction of one full environment of a single trait. The authors’ benchmarked the performance of the PLS for predicting a UT with the Bayesian Genomic Best Linear Unbiased Predictor (GBLUP) method, and in all data sets the UT-PLS method outperformed the UT-GBLUP method by margins between 0% and 228.28% across traits, environments and types of predictors. These results show empirical evidence of the power of PLS methodology for the prediction of future seasons or new environments.

The Multi-Trait Partial Least Square (MT-PLS) regression model is one of the most popular in biological sciences, because it can model complex biological events, it is flexible for considering different factors, and it is unaffected by data collinearity. For this reason, authors suggest that the MT-PLS is a potentially valuable method for modeling high-dimensional biological data (as derived from genomics, proteomics and peptidomics) (Palermo et al., 2009). MT-PLS can model multiple responses, while efficiently dealing with multicollinearity. This model is also used for variable selection as a process to discover the most relevant features. MT-PLS has been successful in biological research because many datasets contain observations of multiple correlated traits. Unlike single-trait association analysis, which cannot extract additional information from correlated traits, joint association analysis like MT-PLS explicitly uses the correlation structure among such traits. For these reasons, MT-PLS is preferred since this technique achieves greater statistical power for gene detection and in terms of prediction performance, it is expected to achieve improved accuracy.

In the current research, we evaluate the prediction performance of two multi-trait methods, MT-PLS and MT-GBLUP, in the context of leave-one-location-out cross validation. The proposed MT-PLS model is an extension of the UT-PLS recently proposed by Montesinos-López et al. (2022a). The goals of this study are three-fold: 1) benchmark the genomic-enabled prediction power of MT-PLS and UT-GBLUP, 2) compare the prediction performance of these two multi-trait methods (MT-PLS and MT-GBLUP) with their corresponding UT versions (UT-PLS and UT-GBLUP) and 3) evaluate the prediction power of these methods within three different predictor models (E + G, E + G + GE, and G + GE where E = Environment; G = genomic, and GE is genomic × environment interaction) under the leave one location out cross validation.

## Materials and methods

### Bayesian MT-GBLUP model

This model is given in Eq. 1 as:

$$Y=1_{n}\mu^{T}+X_{E}\beta_{E}+Z_{L}g+Z_{EL}\mathrm{gE}+\epsilon$$

where Y is the matrix of phenotypic response variables of order $n\times n_{T}$ and ordered first by environments and then by lines, $n_{T}$ denotes the number of traits, $1_{n}$ is a vector of ones of length n , $\mu^{T}$ is a vector of intercepts for each trait of length $n_{T}$ , T denotes the transpose of a vector or matrix, that is, $\mu ={\left[\mu_{1},\;\ldots ,\;\mu_{n_{T}}\right]}^{T},\;\;X_{E}$ is the design matrix of environments of order n×I , I denotes the number of environments, $\beta_{E}$ is the matrix of beta coefficients for environments with a dimension of $I\times n_{T}$ , $Z_{L}$ is the design matrix of lines of order n×J , J denotes the number of lines, g is the matrix of random effects of lines of order $J\times n_{T}$ distributed as $g∼MN_{J\times n_{T}}\left(0,G,\Sigma_{T}\right)$ , that is, with a matrix-variate normal distribution with parameters M=0 , U=G and $V=\Sigma_{T}$ , G denotes the genomic relationship matrix (VanRaden, 2008) built with marker data of order J×J and $\Sigma_{T}$ is the variance-covariance matrix of traits of order $n_{T}\times n_{T}$ . $\;Z_{EL}$ is the design matrix of the genotype × environment interaction of order n×JI , gE is the matrix of genotype  × environment interaction random effects distributed as $\mathrm{gE}∼MN_{JI\times n_{T}}\left(0,G\;⊗H,\Sigma_{T}\right)$ , where H is a general variance-covariance matrix of environments of order  I×I , and G ⊗H is the Kronecker product of the lth type of kernel matrix of lines and the environmental relationship matrix. ϵ is the residual matrix of dimension $\;n\times n_{T}$ distributed as $\epsilon ∼MN_{n\times n_{T}}\left(0,I_{IJ},R\right)$ , where R is the residual variance-covariance matrix of order $n_{T}\times n_{T}$ . It is important to point out that the H matrix was computed from environmental covariates only for data set 4 and 5, since environmental covariates were available only for these two data sets. For this reason, for the remaining data sets (1, 2, and 3) the H matrix was an identity matrix of order I×I . The UT version of the MT-GBLUP is obtained when the response variable (Y) in place of having $n_{T}$ columns contain only one column, which causes that the number of columns of the matrices $\beta_{E}$ , g and gE are reduced from $n_{T}$ columns to only 1. Furthermore, the vector of intercepts $\left(\mu^{T}\right)$ and variance-covariance matrix of traits **(** $\Sigma_{T}$ of genetic effects and R of residual effects) are reduced to a scalar.

### Multi-trait partial least square method

PLS is a multi-trait regression statistical technique introduced by Wold (1966) in the fields of econometrics and chemometrics. PLS is very efficient for dealing with the p>n problem, i.e., when the number of observations ( n ) is less than the number of explanatory variables ( p ) which many times are highly correlated. The multi-trait version of PLS works for relating a matrix of response variables (Y) of order $n\times n_{T}$ to a set of explanatory variables ( X ) (Wold, 2001; Boulesteix and Strimmer, 2006).

In PLS the regression analysis is not done regressing Y on X **,** but rather regressing Y on T , where T are called the Latent Variables (LVs) latent vectors or X **-**scores, and are obtained iteratively. The basic steps to compute the LVs are:

Step 1 Initialize two matrices, E = X and F = Y . Center and normalize each column of E and F .

Step 2 Form cross product matrix $S=X^{\text{T}}Y$ and perform its singular value decomposition (SVD). The first left and right singular vectors, w and q , are used as weight vectors for X and Y , respectively, to obtain scores t and u :

t=Xw=Ew (1)

u=Yq=Fq (2)

where E and F were initialized as both X and Y , respectively. The X scores t are often normalized:

$$t=t/\sqrt{t^{T}t}$$ (3)

The Y scores u are not necessary in the regression but are often saved for interpretation purposes.

Step 3 Next, X and Y loadings are obtained by regressing against the same vector t :

$$p=E^{T}t$$ (4)

$$q=F^{T}t$$ (5)

Step 4 Having extracted the first latent vector and corresponding loading vectors, the matrices E and F are deflated by subtracting information related to the first latent vector. This produces deflated matrices $E_{n+1}$ and $F_{n+1}$ as shown in the calculations below.

$$E_{n+1}=E_{n}-\;tp^{T}$$ (6)

$$F_{n+1}=F_{n}-\;tq^{T}$$ (7)

Step 5 Calculate the cross-product matrix of $E_{n+1}$ and $F_{n+1}$ as was done in Step 2. With this new cross-product matrix, repeat steps 3 and 4 and save the resulting w , t , p and q vectors to form the next columns of matrices **W**, **T**, **P**, and **Q**, respectively. This yields the next component. After this, we continue the above steps until the deflated matrices are empty or the necessary number of components have been extracted. Then the algorithm stops.

It is important to point out that the columns of matrix W cannot be compared directly: they are derived from successively deflated matrices E and F as can be seen in the previous five steps. For this, reason after having all the columns of W **,** we compute R as:

$$R=W{\left(P^{T}W\right)}^{-1}$$ (8)

Finally, using R we can compute the latent variables, which are related to the original X matrix as:

T=XR (9)

Next, since we regress Y on T , the resulting beta coefficients are $b={\left(T^{T}T\right)}^{-1}T^{T}Y$ . However, to convert these back to the realm of the original variables (X) we pre-multiply with matrix R the beta coefficients (b) , since T=XR,

B=R b (10)

To obtain better performance of the PLS method, only the first a components are used. Since regression and dimension reduction are performed simultaneously, all B , T , W , P , and Q are part of the output. Both X and Y are considered when calculating the LVs in T . Moreover, they are defined so that the covariance between the LVs and the matrix of response variables is maximized. Finally, predictions for new data $\left(X_{\mathrm{new}}\right)$ should be done with:

$${\hat{Y}}_{\mathrm{new}}=X_{\mathrm{new}}B=X_{\mathrm{new}}\mathrm{Rb}=T_{\mathrm{new}}b$$ (11)

where $T_{\mathrm{new}}=X_{\mathrm{new}}R$ . Usually, the optimal number of components need to be determined by cross-validation. We used the root means squared error of prediction (RMSEP), which was minimized with 10-fold cross-validation in the training data set and for each value of LV (Mevik and Cederkvist, 2004).

The input used under MT-PLS is the concatenation of the following three augmented matrices: $X_{\text{L}}L_{E},X_{\text{g}}L_{g}$ and $X_{\text{gL}}\left(L_{E}⊗L_{g}\right)$ , which belong to environments, genotypes, and GE components, respectively. It is important to note that the design matrices (dummy variables) of environments $\left(X_{\text{L}}\right)$ , genotypes $\left(X_{\text{g}}\right)$ and GE interaction $\left(X_{\text{gL}}\right)$ were computed first. Then $L_{g}$ and $L_{E}$ were computed. $L_{g}$ denotes the square root of the genomic relationship matrix G , while $L_{E}$ denotes the square root of the environmental relationship matrix H **.** The reason why G and H matrices are square root is because under PLS framework we cannot directly input these covariance matrices as done under the MT-GBLUP model. Also, since the PLS method only requires a unique matrix of inputs (predictors) it is necessary to concatenate all the terms that we suspect are related to the response variables. It is also important to point out that the implementation of both MT models (GBLUP and PLS) was done in the R statistical software **(** R Core Team, 2022), but the MT- GBLUP was done with BGLR library (Pérez and de los Campos, 2014), while the MT-PLS was done with the pls library (Mevik and Wehrens, 2007). Furthermore, here the H matrix was computed from environmental covariates only for data sets 4 and 5, since only for these two data sets environmental covariates were available. The UT-PLS model is obtained when the response variable (Y) is reduced from a matrix to a vector, but the algorithm for all the computations is the same.

### Data sets

#### Datasets 1. Elite wheat yield trial years 2013–2014

These datasets belong to elite yield trials (EYT) from 2013–2014, from the International Maize and Wheat Improvement Center (CIMMYT) that were established in four different cropping seasons within four environments. A total of 776 lines were examined. An alpha-lattice experimental design was used, and the lines were sown in 39 trials, with 28 lines each and 2 checks in 6 blocks with 3 replications. In this study, four traits were evaluated for each line in each environment: days to heading, computed as the number of days from germination to 50% spike emergence (DTHD), days to maturity, computed as the number of days from germination to 50% physiological maturity or the loss of the green color in 50% of the spikes (DTMT), and plant height and grain yield (GY). For more details about the data sets as well as BLUEs computation, Juliana et al. (2018).

For dataset 1, four environments were evaluated. The environments studied were bed planting with five irrigations (Bed5IR), early heat (EHT), flat planting with five irrigations (Flat5IR), and late heat (LHT). Genotyping-by-sequencing (GBS) was used to gather the genome-wide markers of the 776 lines (Elshire et al., 2011; Poland et al., 2012) obtained at Kansas State University using an Illumina HiSeq2500. From an initial set of 34,900 markers, after filtering, 2,038 markers were used. LinkImpute (Money et al., 2015) was used for the imputation of missing marker data and implemented in TASSEL (Bradbury et al., 2007), version 5. Only 2,515 lines were used in this study because lines missing more than 50% of data were removed.

### Dataset 2. Groundnut data

This data set was provided by Pandey et al. (2020) and contains phenotypic and genotypic information for 318 lines and four environments. In the present study we assessed the prediction performance of the following four traits: seed yield per plant (SYPP), pods per plant (NPP), pod yield per plant (PYPP) and yield per hectare (YPH). The environments were denoted as: Aliyarnagar_Rainy 2015 (ENV1); Jalgoan_Rainy 2015 (ENV2); ICRISAT_Rainy 2015 (ENV3); ICRISAT Post-Rainy 2015 (ENV4).

The dataset contained a total of 1272 assessments and is balanced since each line is included once in each environment. For each line, 8,268 single nucleotide polymorphisms (SNP) markers (coded with 0, 1 and 2) were available after quality control.

### Dataset 3. Disease data

In this data set, 438 wheat lines with three disease traits were measured 1) PTR, for *Pyrenophora tritici-repentis* (PTR) that causes yellow spot, also known as yellow leaf spot, tan spot, yellow leaf blotch or helminthosporiosis. 2) SN, for *Parastagonospora nodorum* (SN), is a major fungal pathogen of wheat fungal taxon that includes plant pathogens affecting the leaves and other parts of the plants.and 3) SB denotes *Bipolaris sorokiniana* (SB), which causes seedling diseases, common root rot and spot blotch of several crops such as barley and wheat. These 438 lines were evaluated in the greenhouse for several replicates. The replicates were considered as environments (Env1, Env2, Env3, Env4, Env5, and Env6). For the three traits measured, the total number of observations were 438 ×6 = 2628.

DNA samples were genotyped using 67,436 SNPs. For a given marker, the genotype for each line was coded as the number of copies of a designated marker-specific allele carried by the line (absence = zero and presence = one). SNP markers with unexpected heterozygous genotypes were recoded as either AA or BB. Markers with over 15% missing values and/or MAF <0.05 were removed. A total of 11,617 SNPs were available for analysis after quality control and imputation.

### Dataset 4. Indica

This dataset contains information on the phenotypic performance of four traits (GY = Grain Yield, PHR = Percentage of Head Rice Recovery, GC = percentage of Chalky Grain, PH = Plant Height) of rice and was reported by Monteverde et al. (2019) for three environments in 2010, 2011 and 2012. For each year, 327 lines were evaluated and environmental covariates were measured at each developmental stage: vegetative, reproductive, and maturation. The following 18 environmental covariates were also evaluated: 1) *ThermAmp,* average of daily thermal amplitude calculated as max temperature (°C)—min temperature (°C), 2) *RelSun,* the relative sunshine duration (%) computed as the quotient between the real duration of the brightness of the sun and the possible geographical or topographic duration, 3) *SolRad,* solar radiation (cal/cm2/day) calculated using Armstrong’s formula, 4) *EfPpit,* effective precipitation (mm) computed as the average of daily precipitation in mm added and stored in the soil, 5) *DegDay,* the mean of daily average temperature minus 10°C, 6) *RelH*, relative humidity (hs) computed as the sum of daily amount of hours (0hs–24hs) where the relative humidity was equal to 100%, 7) *PpitDay*, the precipitation day computed as the sum of days when it rained, 8) *MeanTemp*, the mean of temperature (°C) over 24hs (0–24 hs), 9) *AvTemp*, the average temperature (°C) calculated as daily (Max + Min)/2, (10) *MaxTemp*, the average of maximum daily temperature (°C), 11) *MinTemp*, the average of minimum daily temperature (°C), 12) *TankEv*, tank water evaporation (mm) computed as the amount of evaporated water under the influence of sun and wind, 13) *Wind*, wind speed (2 m/km/24 hs) computed as the distance covered by wind (in km) over 2 m height in 1 day, 14) *PicheEv*, the piche evaporation (mm) computed as the amount of evaporated water without the influence of the sun, 15) *MinRelH*, the minimum relative humidity (%) computed as the lowest value of relative humidity for the day, 16) *AccumPpit*, the daily accumulated precipitation (mm), 17) *Sunhs*, the sunshine duration computed as the sum of total hours of sunshine per day, and 18) *MinT15*, the minimum temperature below 15° computed as the sum of the days when the minimum temperature was below 15.

The total number of assessments in this balanced data set is 981 since each line is included once in each environment. After quality control, markers for 16,383 SNPs remained and were coded as 0, 1, and 2.

### Dataset 5. Japonica

Monteverde et al. (2019) reported this rice data set, belonging to the tropical Japonica population with the same four traits (GY = Grain Yield, PHR = Percentage of Head Rice Recovery, GC = percentage of Chalky Grain, PH = Plant Height) as for the Indica population (data set 6) but over the course of 5 years (2009, 2010, 2011, 2012 and 2013). The lines evaluated were 93, 292, 316, 316, and 134 lines for 2009, 2010, 2011, 2012, and 2013, respectively. The same 54 environmental covariates measured in the Indica data set (Data set 4) were evaluated. In this set, 1051 assessments were evaluated in the five environments. A total of 320 lines and 6,383 SNP markers remained after quality control, coded as 0, 1, and 2.

### Metrics for evaluation of prediction accuracy

In each of the five datasets, the leave one environment out cross-validation was implemented (Montesinos-López et al., 2022b). For this reason, I−1 environments were assigned to the training set and the remaining were assigned to the testing set, until each of the I environments was tested once. The MT-GBLUP model *1*) did not require a tuning process, but in the MT-PLS model we tuned the number of principal components using five nested cross-validations dividing the training set (information of the I−1 environments) into the inner training set (80% of the training) and validation (tuning) set (20% of the training) in each of the five folds. Of the five folds, one was used as the validation set and the remaining four as inner training. Next, the average of the five validation folds was reported as the metric of prediction performance to select the optimal hyperparameter (number of principal components) in the MT-PLS model (Montesinos-López et al., 2022b). Then, using this optimal number of hyperparameters, the MT-PLS model was refitted with the whole training set (the I−1 environments) and finally, the prediction of each testing set (a full environment) was obtained. The normalized root mean square error is represented as $\left(NRMSE=\frac{RMSE}{\bar{y}}\right)$ , where $RMSE=\sqrt{\frac{1}{T}(\overset{T}{\underset{i=1}{\sum }}{\left(y_{i}-\hat{f}(x_{i})\right)}^{2}}$ , with $y_{i}\;$ denoting the observed value i , while $\hat{f}\left(x_{i}\right)$ represents the predicted value for observation *i* (*i*=1...*n*) used as a metric to evaluate the prediction accuracy. To compare both models in terms of NRMSE, the relative efficiency was computed as

$$RE_{NRMSE}=\frac{NRMSE_{MT\_GBLUP}}{NRMSE_{MT\_PLS}}$$

where $NRMSE_{MT\_GBLUP}$ and $NRMSE_{MT\_PLS}$ denote the NRMSE under the MT-GBLUP and MT-PLS models, respectively. When $RE_{NRMSE}>1,\;$ the best prediction performance in terms of NRMSE was obtained using the MT PLS method, but when $RE_{NRMSE}<1,$ the MT-GBLUP method was superior in terms of prediction accuracy, when $RE_{NRMSE}=1,$ both methods were equally efficient. Also, to compare the prediction performance for each model, we computed the relative efficiency between the NRMSE obtained under the UT and MT models. This computation was done as

$$RE_{NRMSE}=\frac{NRMSE_{UT}}{NRMSE_{MT}}$$

where $NRMSE_{MT}$ denotes the NRMSE under an MT model, and $NRMSE_{UT}$ denotes the NRMSE under a UT model. Now if $RE_{NRMSE}>1,\;$ the best prediction performance in terms of NRMSE was obtained using the MT model, but when $RE_{NRMSE}<1,$ the UT model was superior in terms of prediction accuracy. When $RE_{NRMSE}=1,$ both models were equally efficient. Note that the observed and predicted values are in the same scale under both models (MT-GBLUP and MT-PLS) for this reason the comparison between models using the NRMSE is valid. We used only the NRMSE since it is one of the most appropriate metrics for comparing when the response variables are in different scales, since it is not dependent on the effect of the scale of the traits. However, even though there are other metrics that can be used for the same task in this paper we only focused in this metric, to capture the essential behavior of both models (MT-GBLUP and MT-PLS).

### Data availability and Supplementary Tables

The data files and the Supplementary Tables can be found in link https://hdl.handle.net/11529/10548705. The phenotypic and genotypic data for the 5 datasets are in EYT_1.RDATA (Dataset 1), Groundnut.RData (Dataset 2), Disease.RData (Dataset 3), Indica.RData (Dataset 4), and Japonica.RData (Dataset 5). Supplementary Tables S1 with the genomic prediction accuracy results of the different data sets are shown in Supplementary Tables SA1,A2 for Dataset 1, Supplementary Tables SB1,B2 for Dataset 2, Supplementary Tables SC1,C2 for Dataset 3, Supplementary Tables SD1,D2 for Dataset 4, and Supplementary Tables SE1,E2 for Dataset 5.

## Results

**FIGURE 1:**
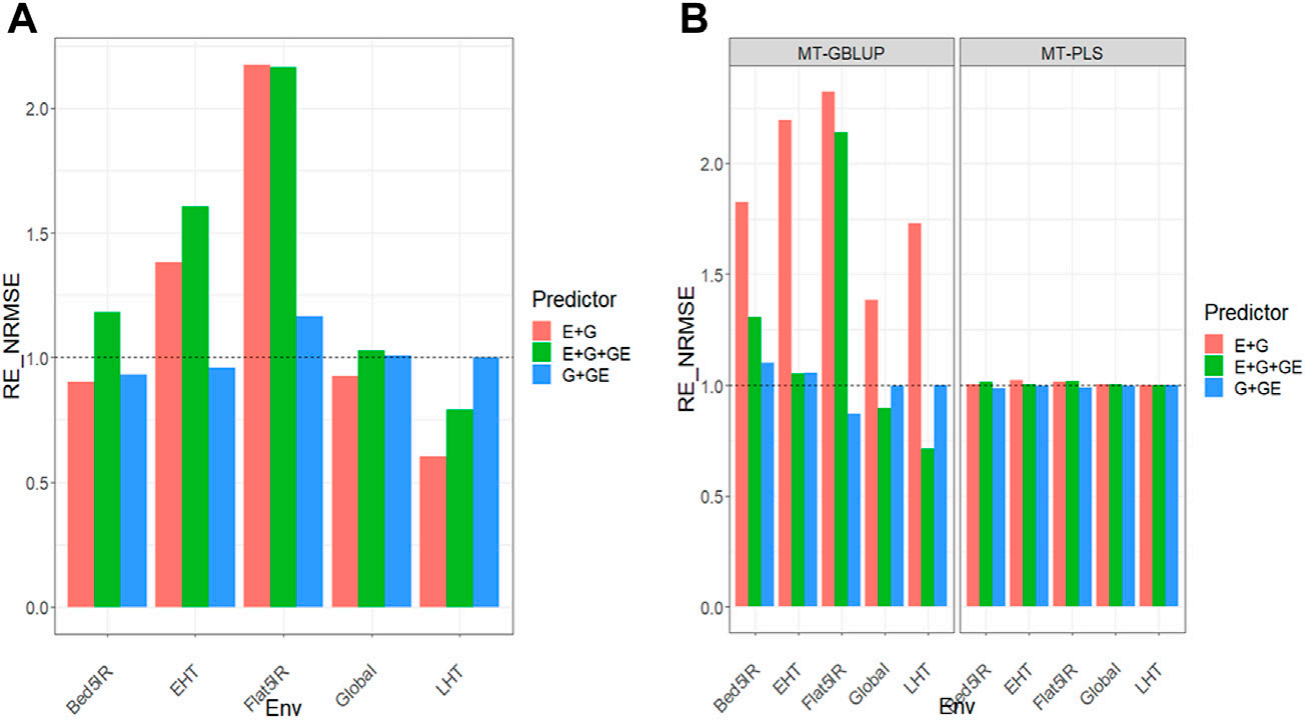
**(A)** Relative efficiency in terms of normalized root mean square error (RE_NRMSE) computed by dividing the NRMSE under the multi-trait best linear unbiased predictor model (MT-GBLUP) by the NRMSE of the multi-trait partial least square regression method (MT-PLS). Prediction performance is reported across traits for each environment and across environments (Global) in dataset 1 (EYT_1), also with three predictors (E + G, E + G + GE and G + GE). When the RE_NRMSE>1 the MT-PLS outperform in terms of prediction performance (lower NRMSE) the MT-GBLUP method. **(B)** Relative efficiency in terms of normalized root mean square error (RE_NRMSE) computed by dividing the NRMSE of either the UT best linear unbiased predictor model (UT-GBLUP) or UT partial least square regression method (UT-PLS), by the NRMSE of either MT-GBLUP or MT-PLS method. Prediction performance is reported across traits for each environment and across environments (Global) in dataset 1 (EYT_1), also with three predictors (E + G, E + G + GE and G + GE). When the RE_NRMSE>1 the multi-trait method outperform in terms of prediction performance (lower NRMSE) the UT method.

**FIGURE 2:**
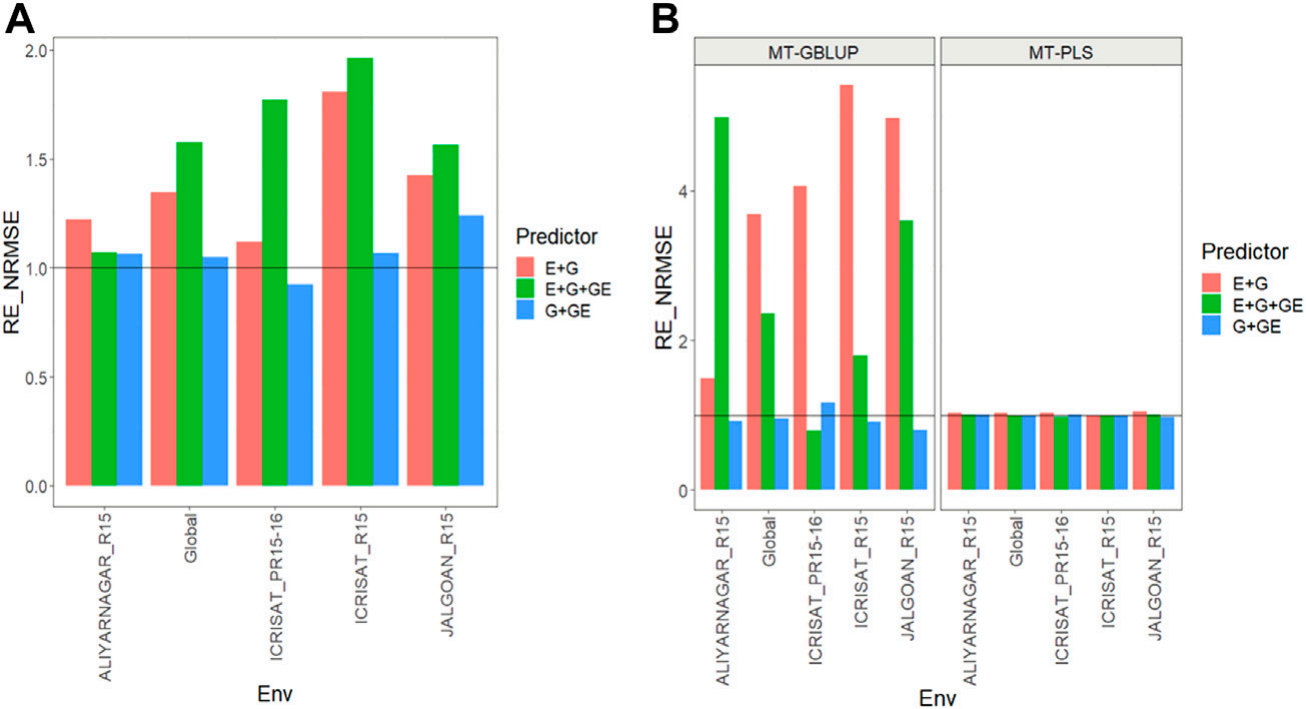
**(A)** Relative efficiency in terms of normalized root mean square error (RE_NRMSE) computed by dividing the NRMSE under the multi-trait best linear unbiased predictor model (MT-GBLUP) by the NRMSE of the multi-trait partial least square regression method (MT-PLS). Prediction performance is reported across traits for each environment and across environments (Global) in dataset 2 (Groundnut), also with three predictors (E + G, E + G + GE and G + GE). When the RE_NRMSE>1 the MT-PLS outperform in terms of prediction performance (lower NRMSE) the MT-GBLUP method. **(B)** Relative efficiency in terms of normalized root mean square error (RE_NRMSE) computed by dividing the NRMSE of either the UT best linear unbiased predictor model (UT-GBLUP) or UT partial least square regression method (UT-PLS), by the NRMSE of either MT-GBLUP or MT-PLS method. Prediction performance is reported across traits for each environment and across environments (Global) in dataset 2 (Groundnut), also with three predictors (E + G, E + G + GE and G + GE). When the RE_NRMSE>1 the multi-trait method outperform in terms of prediction performance (lower NRMSE) the UT method.

**FIGURE 3:**
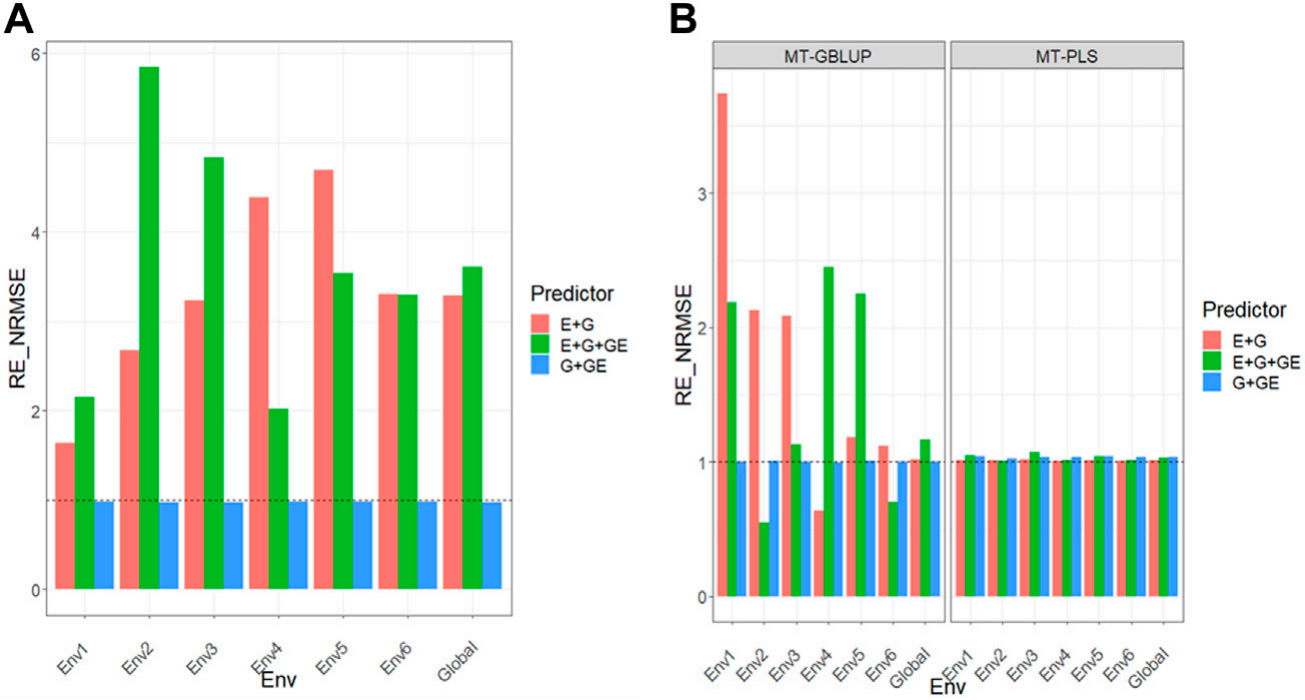
**(A)** Relative efficiency in terms of normalized root mean square error (RE_NRMSE) computed by dividing the NRMSE under the multi-trait best linear unbiased predictor model (MT-GBLUP) by the NRMSE of the multi-trait partial least square regression method (MT-PLS). Prediction performance is reported across traits for each environment and across environments (Global) in dataset 3 (Disease), also with three predictors (E + G, E + G + GE and G + GE). When the RE_NRMSE>1 the MT-PLS outperform in terms of prediction performance (lower NRMSE) the MT-GBLUP method. **(B)** Relative efficiency in terms of normalized root mean square error (RE_NRMSE) computed by dividing the NRMSE of either the UT best linear unbiased predictor model (UT-GBLUP) or UT partial least square regression method (UT-PLS), by the NRMSE of either MT-GBLUP or MT-PLS method. Prediction performance is reported across traits for each environment and across environments (Global) in dataset 3 (Disease), also with three predictors (E + G, E + G + GE and G + GE). When the RE_NRMSE>1 the multi-trait method outperform in terms of prediction performance (lower NRMSE) the UT method.

**FIGURE 4:**
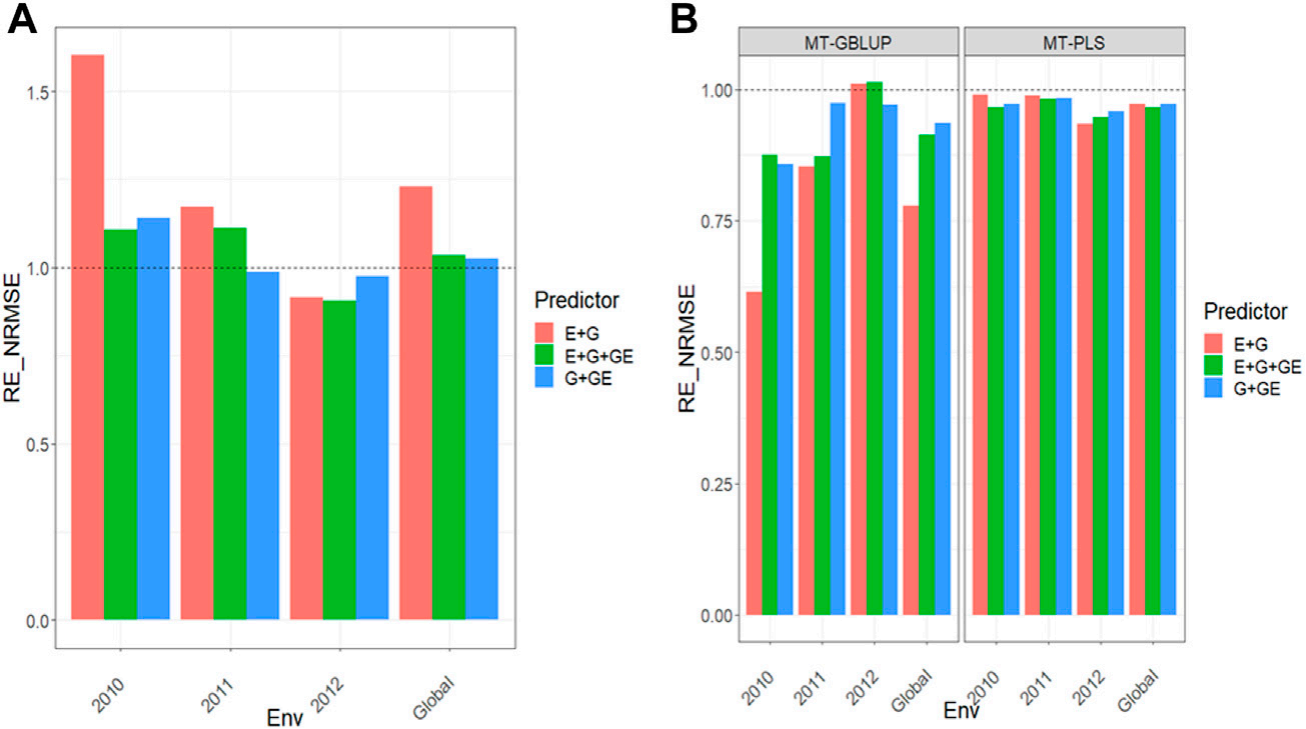
**(A)** Relative efficiency in terms of normalized root mean square error (RE_NRMSE) computed by dividing the NRMSE under the multi-trait best linear unbiased predictor model (MT-GBLUP) by the NRMSE of the multi-trait partial least square regression method (MT-PLS). Prediction performance is reported across traits for each environment and across environments (Global) in dataset 4 (Indica), also with three predictors (E + G, E + G + GE and G + GE). When the RE_NRMSE>1 the MT-PLS outperform in terms of prediction performance (lower NRMSE) the MT-GBLUP method. **(B)** Relative efficiency in terms of normalized root mean square error (RE_NRMSE) computed by dividing the NRMSE of either the UT best linear unbiased predictor model (UT-GBLUP) or UT partial least square regression method (UT-PLS), by the NRMSE of either MT-GBLUP or MT-PLS method. Prediction performance is reported across traits for each environment and across environments (Global) in dataset 4 (Indica), also with three predictors (E + G, E + G + GE and G + GE). When the RE_NRMSE>1 the multi-trait method outperform in terms of prediction performance (lower NRMSE) the UT method.

**FIGURE 5:**
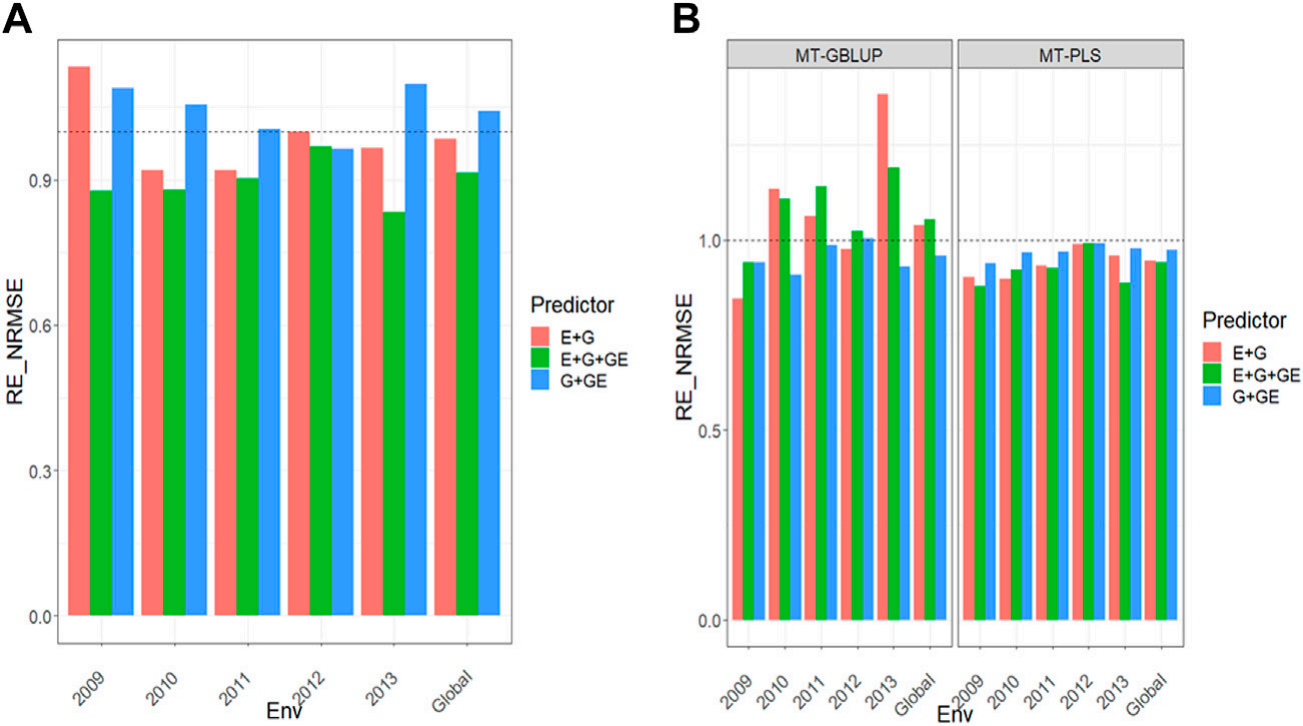
**(A)** Relative efficiency in terms of normalized root mean square error (RE_NRMSE) computed by dividing the NRMSE under the multi-trait best linear unbiased predictor model (MT-GBLUP) by the NRMSE of the multi-trait partial least square regression method (MT-PLS). Prediction performance is reported across traits for each environment and across environments (Global) in dataset 5 (Japonica), also with three predictors (E + G, E + G + GE and G + GE). When the RE_NRMSE>1 the MT-PLS outperform in terms of prediction performance (lower NRMSE) the MT-GBLUP method. **(B)** Relative efficiency in terms of normalized root mean square error (RE_NRMSE) computed by dividing the NRMSE of either the UT best linear unbiased predictor model (UT-GBLUP) or UT partial least square regression method (UT-PLS), by the NRMSE of either MT-GBLUP or MT-PLS method. Prediction performance is reported across traits for each environment and across environments (Global) in dataset 5 (Japonica), also with three predictors (E + G, E + G + GE and G + GE). When the RE_NRMSE>1 the multi-trait method outperform in terms of prediction performance (lower NRMSE) the UT method.

All the results are displayed in Figures 1A,B for Dataset 1, Figures 2A,B for Dataset 2, Figures 3A,B for Dataset 3, Figures 4A,B for Dataset 4, and Figures 5A,B for Dataset 5. Detail information for the results of the 5 data sets can also be found on the Supplemental Tables.

### Dataset 1. EYT_1

Results are displayed in Figures 1A,B and further details are given in Supplementary Tables SA1,A2.

### MT-GBLUP versus MT-PLS

We observed that the relative efficiencies in terms of NRMSE of the MT-GBLUP method and MT-PLS method for predictor **E + G** were 0.935, 1.381, 2.202, 0.625, and 0.921 for environments Bed5IR, EHT, Flat5IR, and LHT and across environments (Global), respectively. The MT-PLS method outperformed the MT-GBLUP method only for environments EHT and Flat5IR by (1.381–1) ×100= 38.1% EHT and (2.202–1) ×100= 120.2%, respectively. In the case of the **E + G + GE** predictor, the observed relative efficiencies were 1.000 (Bed5IR), 1.475 (EHT), 2.118 (Flat5IR), 0.813 (LHT) and 1.040 (Global). This indicates that MT-PLS had better prediction accuracy than MT-GBLUP in most environments by 47.5% (EHT), 111.8% (Flat5IR) and 4.0% (Global). For the **G + GE** predictor, the relative efficiencies observed were 0.936 (Bed5IR), 0.962 (EHT), 1.134 (Flat5IR), 1.003 (LHT) and 1.007 (Global). Only for Flat5IR, LHT and across environments, MT-PLS had better prediction performance than MT-GBLUP by 13.4% (Flat5IR), 0.3% (LHT) and 0.7% (Global). Figure 1A. For more details, Supplementary Table SA1.

### Uni-trait versus multi-trait

We observed that the relative efficiencies for two GBLUP methods (MT and UT), in terms of NRMSE for predictor **E + G**, were 1.833 (Bed5IR), 2.016 (EHT), 1.120 (Flat5IR), 1.219 (LHT) and 1.417 (Global). The multi-trait method outperformed the UT method in every environment by 83.3% (Bed5IR), 101.6% (EHT), 12.0% (Flat5IR), 21.9% (LHT) and 41.7% (Global). For **E + G + GE**, the observed relative efficiencies were 0.916 (Bed5IR), 0.760 (EHT), 1.929 (Flat5IR), 0.466 (LHT) and 0.875 (Global). Multi-trait outperformed UT only in Flat5IR environment by 92.9%. In **G + GE**, the relative efficiencies were 1.085 (Bed5IR), 1.050 (EHT), 0.872 (Flat5IR), 0.999 (LHT) and 0.996 (Global), meaning the UT method had better prediction performance than the multi-trait method only in Bed5IR and EHT environments by 8.5% (Bed5IR), 5.0% (EHT).

Regarding both implementations under the **PLS** (MT and UT) method, the observed relative efficiencies in terms of NRMSE for predictor **E + G** were 1.004 (Bed5IR), 1.022 (EHT), 1.012 (Flat5IR), 1.001 (LHT) and 1.004 (Global), indicating that the multi-trait PLS method had better prediction performance than the UT-PLS by 0.4% (Bed5IR), 2.2% (EHT), 1.2% (Flat5IR), 0.1% (LHT) and 0.4% (Global). For the **E + G + GE** predictor, the observed relative efficiencies were 1.011 (Bed5IR), 0.999 (EHT), 1.021 (Flat5IR), 1.000 (LHT) and 1.004 (Global). The multi-trait PLS method outperformed the UT PLS method in every environment except the EHT and LHT environments by 1.1% (Bed5IR), 2.1% (Flat5IR) and 0.4% (Global). Finally, for the **G + GE** predictor, the observed relative efficiencies were 0.987 (Bed5IR), 0.996 (EHT), 0.987 (Flat5IR), 1.001 (LHT) and 0.996 (Global), so the MT-PLS method outperformed UT- PLS only in the LHT environment by 0.1% Figure 1B. For more details, Supplementary Table SA2 Results show that MT-PLS overcame the genomic-enabled prediction accuracy of MT-GBLUP, and MT-GBLUP is more precise than UT-GBLUP; however MT-PLS and UT-LS yielded similar genomic-enabled prediction accuracy.

### Dataset 2. Groundnut

Results are shown in Figures 2A,B and further details are in Supplementary Tables SB1,B2.

### MT-GBLUP versus MT-PLS

We observed that the relative efficiencies in terms of NRMSE of the MT-GBLUP method and MT-PLS method for predictor **E + G** were 1.230, 1.110, 1.800, 1.424, and 1.343 for environments ALIYARNAGAR_R15, ICRISAT_PR15-16, ICRISAT_R15, JALGOAN_R15 and across environments (Global), respectively. So the MT-PLS method outperformed MT-GBLUP method by 23.0% in ALIYARNAGAR_R15, 11.0% in ICRISAT_PR15-16, 80.0% in ICRISAT_R15, 42.4% in JALGOAN_R15 and 34.3% across environments. In the case of the **E + G + GE** predictor, the observed relative efficiencies were 1.075 (ALIYARNAGAR_R15), 1.774 (ICRISAT_PR15-16), 1.980 (ICRISAT_R15), 1.566 (JALGOAN_R15) and 1.600 (Global), indicating that MT-PLS had a better prediction accuracy than MT-GBLUP by 7.5% (ALIYARNAGAR_R15), 77.4% (ICRISAT_PR15-16), 98.0% (ICRISAT_R15), 56.6% (JALGOAN_R15) and 60.0% (Global). For the **G + GE** predictor, the relative efficiencies observed were 1.056 (ALIYARNAGAR_R15), 0.877 (ICRISAT_PR15-16), 1.062 (ICRISAT_R15), 1.238 (JALGOAN_R15) and 1.040 (Global), meaning that MT-PLS outperformed MT-GBLUP in every environment except ICRISAT_PR15-16 by 5.6% (ALIYARNAGAR_R15), 6.2% (ICRISAT_R15), 23.8% (JALGOAN_R15) and 4.0% (Global) Figure 2A (Supplementary Table SB1).

### Uni-trait versus multi-trait

We observed that the relative efficiencies for **MT** versus **UT** methods both under a GBLUP framework, in terms of NRMSE for predictor **E + G**, were 1.614 (ALIYARNAGAR_R15), 4.043 (ICRISAT_PR15-16), 3.004 (ICRISAT_R15), 4.581 (JALGOAN_R15) and 3.346 (Global), indicating that the multi-trait method outperformed the UT method in every environment by 61.4% (ALIYARNAGAR_R15), 304.3% (ICRISAT_PR15-16), 200.4% (ICRISAT_R15), 358.1% (JALGOAN_R15) and 234.6% (Global). With **E + G + GE,** also under a GBLUP framework, the observed relative efficiencies were 4.784 (ALIYARNAGAR_R15), 0.702 (ICRISAT_PR15-16), 1.881 (ICRISAT_R15), 2.849 (JALGOAN_R15) and 2.127 (Global), so MT outperformed UT by 378.4% (ALIYARNAGAR_R15), 88.1% (ICRISAT_R15), 184.9% (JALGOAN_R15) and 112.7% (Global). In **G + GE**, under the GBLUP framework, the relative efficiencies were 0.920 (ALIYARNAGAR_R15), 1.156 (ICRISAT_PR15-16), 0.901 (ICRISAT_R15), 0.794 (JALGOAN_R15) and 0.948 (Global), meaning that the MT method had a better prediction performance than UT only in the ICRISAT_PR15-16 environment by 15.6%.

Regarding the comparison between MT and UT methods under a **PLS** framework, the observed relative efficiencies in terms of NRMSE for predictor **E + G** were 1.025 (ALIYARNAGAR_R15), 1.027 (ICRISAT_PR15-16), 0.997 (ICRISAT_R15), 1.049 (JALGOAN_R15) and 1.025 (Global), indicating that the multi-trait method had a better prediction performance by 2.5% (ALIYARNAGAR_R15), 2.7% (ICRISAT_PR15-16), 4.9% (JALGOAN_R15) and 2.5% (Global). For the **E + G + GE** predictor, also under a PLS framework, the observed relative efficiencies were 1.008 (ALIYARNAGAR_R15), 0.962 (ICRISAT_PR15-16), 0.997 (ICRISAT_R15), 1.003 (JALGOAN_R15) and 0.989 (Global), meaning that the multi-trait method outperformed the UT method only in the ALIYANAGAR_R15 and JALGOAN_R15 environments by 0.8% and 0.3%, respectively. For the **G + GE** predictor, the observed relative efficiencies were 1.012 (ALIYARNAGAR_R15), 1.007 (ICRISAT_PR15-16), 0.998 (ICRISAT_R15), 0.971 (JALGOAN_R15) and 0.998 (Global), which means that the multi-trait method (MT-PLS) outperformed UT only in the ALIYARNAGAR_R15 environment by 0.7% Figure 2B (Supplementary Table SB2). In summary, while MT-PLS gave better genomic-enabled prediction accuracy than MT-GBLUP, and MT-GBLUP is more precise than UT-GBLUP, and MT-PLS, UT-PLS had similar genomic-enabled prediction accuracy.

### Dataset 3. Disease

Results are given in Figures 3A,B and further details are in Supplementary Tables SC1,C2.

### MT-GBLUP versus MT-PLS

We observed that the relative efficiencies in terms of NRMSE computed with the MT-GBLUP results divided by the MT-PLS results for predictor **E + G** were 1.634, 2.755, 3.252, 4.359, 4.498, 3.207, and 3.300 for environments Env1, Env2, Env3, Env4, Env5, Env6 and across environments (Global), respectively. This indicates that MT-PLS method outperformed the MT-GBLUP method in all the environments by 63.4% (Env1), 175.5% (Env2), 225.2% (Env3), 335.9 (Env4), 349.8% (Env5), 220.7% (Env6) and 230.0 (Global). In the case of the **E + G + GE** predictor, the observed relative efficiencies were 2.166 (Env1), 5.844 (Env2), 4.821 (Env3), 2.051 (Env4), 3.453 (Env5), 3.498 (Env6) and 3.640 (Global), revealing that MT-PLS had better prediction accuracy than MT-GBLUP in every environment by 116.6% (Env1), 484.4% (Env2), 382.1% (Env3), 105.1% (Env4), 245.3% (Env5), 249.8% (Env6) and 264.0% (Global). For the **G + GE** predictor, the relative efficiencies observed were 0.980 (Env1), 0.970 (Env2), 0.969 (Env3), 0.978 (Env4), 0.977 (Env5), 0.982 (Env6) and 0.975 (Global), meaning that MT-PLS was outperformed by MT-GBLUP in all the environments by 2.0% (Env1), 3.0% (Env2), 3.1% (Env3), 2.2% (Env4), 2.3% (Env5), 1.8% (Env6) and 2.5% (Global) Figure 3A (Supplementary Table SC1).

### Uni-trait versus multi-trait

We observed that the relative efficiencies in terms of NRMSE when comparing UT versus MT models in a GBLUP framework, under predictor **E + G**, were 3.142 (Env1), 1.211 (Env2), 1.369 (Env3), 0.348 (Env4), 0.749 (Env5), 0.830 (Env6) and 1.031 (Global), indicating that the multi-trait method outperformed the UT method in only Env1 (214.2%), Env2 (21.1%), Env3 (36.9%) and Global (3.1%). For **E + G + GE,** also under a GBLUP framework, the observed relative efficiencies were 2.155 (Env1), 0.622 (Env2), 1.191 (Env3), 1.559 (Env4), 1.722 (Env5), 0.471 (Env6) and 1.135 (Global), so the multi-trait model outperformed the UT model only in some environments by 115.5% (Env1), 19.1% (Env3), 55.9% (Env4), 72.2% (Env5) and 13.5% (Global). When the predictor evaluated was **G + GE**, the relative efficiencies were 1.004 (Env1), 1.005 (Env2), 1.002 (Env3), 0.996 (Env4), 1.006 (Env5), 0.999 (Env6) and 1.002 (Global). This means that the multi-trait method had a better prediction performance than the UT method only in some environments by 0.4% (Env1), 0.5% (Env2), 0.2% (Env3), 0.6% (Env5) and 0.2% (Global).

Regarding the comparison between the UT and MT models under the P**LS** method, the observed relative efficiencies in terms of NRMSE with predictor **E + G** were 1.014 (Env1), 1.014 (Env2), 1.020 (Env3), 1.008 (Env4), 1.012 (Env5), 1.006 (Env6) and 1.012 (Global), so the multi-trait method had better prediction performance than the UT method by 1.4% (Env1), 1.4% (Env2), 2.0% (Env3), 0.8% (Env4), 1.2% (Env5), 0.6% (Env6) and 1.2% (Global). For the **E + G + GE** predictor, also under a PLS framework, the observed relative efficiencies were 1.051 (Env1), 1.009 (Env2), 1.071 (Env3), 1.013 (Env4), 1.047 (Env5), 1.013 (Env6) and 1.034 (Global), indicating that the multi-trait method outperformed the UT method by 5.1% (Env1), 0.9% (Env2), 7.1% (Env3), 1.3% (Env4), 4.7% (Env5), 1.3% (Env6) and 3.4% (Global). Finally, for the **G + GE** predictor, the observed relative efficiencies were 1.044 (Env1), 1.029 (Env2), 1.039 (Env3), 1.044 (Env4), 1.047 (Env5), 1.038 (Env6) and 1.040 (Global). This means that the MT method outperformed the UT by 4.4% (Env1), 2.9% (Env2), 3.9% (Env3), 4.4% (Env4), 4.7% (Env5), 3.8% (Env6) and 4.0% (Global) Figure 3B (Supplementary Table SC2). Results show that while MT-PLS provided better genomic-enabled prediction accuracy than MT-GBLUP, MT-PLS did not improve the genomic-enabled prediction accuracy of UT-PLS.

### Dataset 4. Indica

Results for this data sets can be found in Figures 4A,B and further details are found in Supplementary Tables SD1,D2.

### MT-GBLUP versus MT-PLS

We observed that the relative efficiencies in terms of NRMSE in the comparison between the MT-GBLUP method and MT-PLS method for predictor **E + G** were 1.577, 1.224, 0.915 and 1.231 for environments 2010, 2011, 2012 and across environments (Global), respectively. The MT-PLS method outperformed the MT-GBLUP method in all the environments except in years 2012 by 57.7% (2010), 22.4% (2011) and 23.1% (Global). In the **E + G + GE** predictor, the observed relative efficiencies were 1.091 (2010), 1.134 (2011), 0.904 (2012) and 1.043 (Global), indicating that MT-PLS had a better prediction accuracy than MT-GBLUP in every environment except 2012 by 9.1% (2010), 13.4% (2011) and 4.3% (Global). For the **G + GE** predictor, the relative efficiencies observed were 1.135 (2010), 0.986 (2011), 0.984 (2012) and 1.031 (Global), which means that MT-PLS outperformed MT-GBLUP only in 2010 and across environments (Global) by 13.5% (2010) and 3.1% (Global) Figure 4A (Supplementary Table SD1 **)**.

### Uni-trait versus multi-trait

We observed the relative efficiencies when comparing UT vs. MT models under a GBLUP framework. For predictor **E + G**, the relative efficiencies were 0.607 (2010), 0.773 (2011), 1.016 (2012) and 0.768 (Global), indicating that multi-trait method outperformed the UT method only in environment 2012 by 1.6%. For **E + G + GE**, the observed relative efficiencies were 0.852 (2010), 0.849 (2011), 1.019 (2012), and 0.900 (Global). The multi-trait model outperformed the UT model under the GBLUP framework only in environment 2012 by 1.9%. In **G + GE**, the relative efficiencies were 0.853 (2010), 0.976 (2011), 0.963 (2012) and 0.930 (Global), meaning that the UT method had better prediction performance than the multi-trait method in every environment by 14.7% (2010), 2.4% (2011), 3.7% (2012) and 7.0% (Global). Regarding the comparison between the UT versus the MT model under the **PLS** method, the observed relative efficiencies in terms of NRMSE for predictor **E + G** were 0.993 (2010), 0.987 (2011), 0.937 (2012), and 0.972 (Global). This means that the multi-trait method was outperformed by UT in all the environments by 6.7% (2010), 1.3% (2011), 6.3% (2012) and 2.8% (Global).

For the **E + G + GE** predictor, the relative efficiencies were 0.969 (2010), 0.980 (2011), 0.949 (2012) and 0.966 (Global). The multi-trait method was outperformed by the UT method in every environment by 3.1% (2010), 2.0% (2011), 5.1% (2012) and 3.4% (Global). Finally, for the **G + GE** predictor, the observed relative efficiencies were 0.975 (2010), 0.985 (2011), 0.961 (2012) and 0.974 (Global), revealing that the multi-trait method was outperformed by UT in all the environments by 2.5% (2010), 1.5% (2011), 3.9% (2012) and 2.6% (Global) Figure 4B (Supplementary Table SD2). MT-PLS method gave better genomic-enabled prediction accuracy than MT-GBLUP. While MT-GBLUP was better than UT-GBLUP, MT-PLS did not improve the genomic-enabled prediction accuracy of UT-PLS.

### Dataset 5. Japonica

For this dataset results can be found in Figures 5A,B and other details are in Supplementary Tables E1,E2.

### MT-GBLUP versus MT-PLS

We observed that in the prediction performance of the MT-GBLUP method and MT-PLS method for predictor **E + G**, the relative efficiencies were 1.128, 0.948, 0.926, 0.981, 0.966, and 0.995 for environments 2009, 2010, 2011, 2012, 2013 and across environments (Global), respectively. That is, the MT-PLS method outperformed the MT-GBLUP method only in the 2009 environment by 12.8%. With the **E + G + GE** predictor, the observed relative efficiencies were 0.921 (2009), 1.017 (2010), 0.898 (2011), 0.973 (2012), 0.823 (2013), and 0.948 (Global), indicating that MT-PLS had better prediction accuracy than MT-GBLUP only in 2010 by 1.7%. For the **G + GE** predictor, the relative efficiencies observed were 1.116 (2009), 1.094 (2010), 1.007 (2011), 0.970 (2012), 1.099 (2013), and 1.057 (Global), meaning that MT-PLS outperformed MT-GBLUP in every environment except 2012 by 11.6% (2009), 9.4% (2010), 0.7% (2011), 9.9% (2013), and 5.7% (Global) Figure 5A (Supplementary Table SE1).

### Uni-trait versus multi-trait

The relative efficiencies in terms of NRMSE when comparing UT versus MT models under GBLUP for predictor **E + G** were 0.968 (2009), 1.519 (2010), 1.011 (2011), 0.991 (2012), 1.305 (2013), and 1.166 (Global), indicating that the multi-trait method outperformed the UT method in every environment except 2009 and 2012 by 51.9% (2010), 1.1% (2011), 30.5% (2013), and 16.6% (Global). For the predictor **E + G + GE**, the observed relative efficiencies were 0.887 (2009), 1.043 (2010), 1.116 (2011), 1.002 (2012), 1.186 (2013), and 1.021 (Global). That is, the multi-trait model outperformed the UT model in most environments by 4.3% (2010), 11.6% (2011), 0.2% (2012), 18.6% (2013), and 2.1% (Global), while under predictor **G + GE**, the relative efficiencies were 0.958 (2009), 0.925 (2010), 0.987 (2011), 1.005 (2012), 0.913 (2013), and 0.958 (Global). This shows that the multi-trait method only had better prediction performance than the UT model in environment 2012 by 0.5%. Regarding the prediction performance between the UT and MT models under the **PLS** framework, the observed relative efficiencies in terms of NRMSE for predictor **E + G** were 0.954 (2009), 0.975 (2010), 0.941 (2011), 0.986 (2012), 0.954 (2013), and 0.966 (Global), indicating the multi-trait method was outperformed by the UT model in all the environments by 4.6% (2009), 2.5% (2010), 5.9% (2011), 1.4% (2012), 4.6% (2013), and 3.4% (Global).

For the **E + G + GE** predictor, the observed relative efficiencies were 0.937 (2009), 1.009 (2010), 0.937 (2011), 0.986 (2012), 0.879 (2013), and 0.964 (Global), meaning that the multi-trait method outperformed the UT method only in environment 2010 by 0.9%. Finally, for the **G + GE** predictor, the observed relative efficiencies were 0.977 (2009), 0.992 (2010), 0.969 (2011), 0.989 (2012), 0.973 (2013), and 0.982 (Global). This means that the multi-trait method was outperformed by the UT method in all the environments by 2.3% (2009), 0.8% (2010), 3.1% (2011), 1.1% (2012), 2.7% (2013), and 1.8% (Global) Figure 5B (Supplementary Table SE2). Like the previous findings, the MT-PLS method provided better prediction accuracy than MT-GBLUP and MT-GBLUP better than UT-GBLUP, MT-PLS did not improve the genomic-enabled prediction accuracy of UT-PLS.

## Discussion

Multi-trait models use all the available traits simultaneously and thus are able to capture the correlation between traits. When this correlation between traits is moderate or large, most of the time the prediction performance of MT models is better than that of UT models (Montesinos-López et al., 2016; Montesinos-López et al., 2019a; Montesinos-López et al., 2019b; Montesinos-López et al., 2019c; Montesinos-López et al., 2020). However, most of the MT models are unable to capture complex non-linear patterns. Studies on Bayesian linear and non-linear multi-trait kernel methods for genomic prediction of multi-environment data showed that non-linear Gaussian kernel method outperformed conventional Bayesian Ridge and GBLUP multi-trait linear models (Montesinos-López et al., 2021).

It is important to note that this research evaluated the prediction performance of MT-PLS versus MT-GBLUP. Both models are not new in the context of multivariate analysis; however, the MT-GBLUP is more popular in GS, while the MT-PLS is more popular in the context of chemometrics (i.e., computational chemistry) and the biological sciences. The advantages of multi-trait models are well documented; however, larger data sets require more computing resources, as there are additional parameters that need to be estimated (beta coefficients and genetic and error covariances), which affect the efficiency of genomic prediction. MT-PLS is a novel technique that generalizes and combines features from principal component analysis and multiple regression.

There is much empirical evidence showing that MT-PLS is useful when predicting a set of dependent variables from a large set of independent variables (i.e., inputs), because MT-PLS finds components from the input X that are also relevant for Y . Specifically, MT-PLS searches for a set of latent vectors (principal components) that perform simultaneous decomposition of X and Y with the constraint that these principal components explain as much as possible of the covariance between X and Y . It is followed by a regression step where the decomposition of X is used to predict Y . The main difference between the principal component regression (PCR) and the MT-PLS method is that PCR is an unsupervised method since it considers only the predictors variables (which are interrelated) in the construction of the principal components (applying principal component analysis, PCA on the inputs), whereas MT-PLS is a supervised method since also takes the dependent variables into account (Garthwaite, 1994) for computing the required number of latent variables.

Our results provide relevant empirical evidence that the MT-PLS methodology is very competitive for multi-trait prediction in genomic selection. For example, we found that in most data sets under predictors (E + G and E + G + GE models), MT-PLS outperformed the MT-GBLUP model by large margins: by 349.8% (under predictor E + G), 484.4% (under predictor E + G + GE) and 15.9% (under predictor G + GE) across traits. Also, in this context of multi-trait prediction under the MT-PLS, no large differences were observed in terms of prediction performance under the three predictors examined (E + G; E + G + GE and G + GE), which can be attributed to the PLS methodology not working directly with the independent variables but with a compressed version of the original independent variables, called latent variables. While using the MT-GBLUP model, we observed very large differences in terms of prediction performance among the three predictors (E + G; E + G + GE and G + GE), but in general under the predictor G + GE, we observed stable and low predictions.

It is important to point out that the MT-PLS is very attractive since is very competitive in terms of prediction performance compared with the Bayesian MT-GBLUP. However, more research should be conducted to be able to compare the MT-PLS with other multi-trait models like: 1) the BMTME, which allows researchers to simultaneously model the correlation of lines, traits and environments (Montesinos-López et al., 2019a); 2) conventional multi-trait mixed models (Montesinos-López et al., 2022c); 3) state of the art statistical machine learning methods like random forest (Montesinos-López et al., 2022d) and even deep learning methods (Montesinos-López et al., 2018; 2019c). Nevertheless more benchmarking studies need to be conducted to have a more precise idea of its power in terms of prediction performance in the context of multi-trait analysis. Our results provide empirical evidence that MT-PLS should be considered as a power ally statistical machine learning method for multi-trait prediction in plant breeding.

The MT-PLS methodology is not new in plant breeding, as it has been used for association and prediction studies. For example, Vargas et al. (1998), Vargas et al. (1999) and Crossa et al. (1999) used this methodology for interpreting genotype by environment interaction in maize and wheat. For prediction in GS, this methodology has been used by Monteverde et al. (2019), Colombani et al. (2012) and by Montesinos-López et al. (2022a) for UT predictions.

It is important to highlight that the larger the data set, the more computational resources are required under the MT-PLS model because in order to select the optimal hyperparameters (number of principal components), an inner (nested) cross-validation needs to be implemented to select the optimal number of principal components. However, Silveira et al. (2017) conducted research for selecting the optimal number of principal components in the context of UT-PLS using nested cross-validation as we did and using the degree-of-freedom (DoF) method and they reported that both approaches found the same optimal number of components. This option for selecting the optimal number of principal components should be explored in the context of MT-PLS since significant computational resources can be saved for implementing the MT-PLS method. Choosing the optimal number of components remains a relevant issue for UT and multi-trait PLS applications.

## Conclusions

This research evaluated the prediction performance of the MT-PLS method and compared with the MT-GBLUP method under the leave one environment-out cross-validation. We found that the MT-PLS method is very competitive because in two of the predictors evaluated (E + G; E + G + GE), it significantly outperformed the MT-GBLUP method. However, using the predictor (G + GE), no relevant gain was observed for MT-PLS over MT-GBLUP. When we compared MT-PLS to UT-PLS, no significant gain was observed in terms of prediction performance; however, we found better prediction of MT-GBLUP compared to UT-GBLUP. It is important to point out that to successfully implement the MT-PLS, we needed to implement a nested cross-validation (divide the training set into inner training and validation) to obtain the optimal number of principal components. With this optimal number of principal components inserted into the model using the complete training set, we obtained the predictions for the testing set. However, the tuning process of the MT-PLS is not complex and or time consuming since only one hyperparameter (number of principal components) needs to be tuned. Finally, in this research we provided empirical evidence of the advantages and disadvantages of using the MT-PLS methodology for genomic prediction in the context of multi-trait data.

## Data Availability

The original contributions presented in the study are included in the article/Supplementary Material, further inquiries can be directed to the corresponding authors.
