# Supplementary material for: Multi-trait genome prediction of new environments with partial least squares
Source: Front Genet. 2022 Sep 5;13:966775. doi: 10.3389/fgene.2022.966775 (PMC9483856; doi:10.3389/fgene.2022.966775)
Supplement: Supplementary file 1 [file Table1.pdf]

SUPLEMENTARY TABLE A1.

Prediction performance across traits for each environment and across environments (Global) of **dataset 1** (EYT_1) in terms of mean square error (MSE), normalized root mean square error (NRMSE), Person´s correlation (Cor) and relative efficiency (RE) under three predictors (E+G; environments plus genotypes, E+G+GE, that also contain the genotype by environment interaction and G+GE that contain the genotypes and also the genotype by environment interaction). MSE_P and MSE_G denote the MSE under the MT-PLS and MT-GBLUP method. NRMSE_P and NRMSE_G denote the NRMSE under the MT-PLS and MT-GBLUP method. Cor_P and Cor_G denote the Person´s correlation under the MT-PLS and MT-GBLUP method. RE_MSE, RE_NRMSE and RE_Cor denote the RE computed with the MSE, NRMSE and the Person´s correlation respectively. RE were computed by dividing the prediction performance (with MSE, NRMSE or Cor) of the MT-GBLUP method by the prediction performance of the MT-PLS method.

Predictor	DataSet	Env	MSE_P	NRMSE_P	Cor_P	MSE_G	NRMSE_G	Cor_G	RE_MSE	RE_NRMSE	RE_Cor
E+G+GE	EYT_1	Bed5IR	80.735	2.006	0.594	99.977	2.007	0.691	1.238	1.000	0.860
E+G+GE	EYT_1	EHT	52.010	1.114	0.531	85.011	1.643	0.627	1.635	1.475	0.847
E+G+GE	EYT_1	Flat5IR	11.086	1.064	0.551	40.353	2.254	0.593	3.640	2.118	0.929
E+G+GE	EYT_1	LHT	756.604	6.830	0.504	802.585	5.552	0.518	1.061	0.813	0.973
E+G+GE	EYT_1	Global	225.108	2.753	0.545	256.982	2.864	0.607	1.142	1.040	0.898
E+G	EYT_1	Bed5IR	76.752	1.970	0.646	64.187	1.841	0.676	0.836	0.935	0.956
E+G	EYT_1	EHT	44.315	1.050	0.592	73.228	1.450	0.604	1.652	1.381	0.980
E+G	EYT_1	Flat5IR	14.417	1.196	0.584	120.329	2.634	0.591	8.346	2.202	0.988
E+G	EYT_1	LHT	767.081	6.875	0.504	430.632	4.295	0.515	0.561	0.625	0.979
E+G	EYT_1	Global	225.641	2.773	0.582	172.094	2.555	0.597	0.763	0.921	0.975
G+GE	EYT_1	Bed5IR	81.427	2.021	0.587	71.784	1.892	0.320	0.882	0.936	1.834
G+GE	EYT_1	EHT	217.461	2.319	0.553	201.078	2.232	0.334	0.925	0.962	1.656
G+GE	EYT_1	Flat5IR	67.414	2.186	0.495	79.358	2.480	0.324	1.177	1.134	1.528
G+GE	EYT_1	LHT	817.730	7.140	0.508	821.430	7.158	0.507	1.005	1.003	1.002
G+GE	EYT_1	Global	296.008	3.416	0.536	293.412	3.441	0.371	0.991	1.007	1.445

SUPLEMENTARY TABLE A2.

Prediction performance across traits for each environment and across environments (Global) of **dataset 1** (EYT_1) in terms of mean square error (MSE), normalized root mean square error (NRMSE), Pearson´s correlation (Cor) and relative efficiency (RE) under three predictors (E+G; environments plus genotypes, E+G+GE, that also contain the genotype by environment interaction and G+GE that contain the genotypes and also the genotype by environment interaction) and two models (MT-PLS and MT-GBLUP). MSE_U and MSE_M denote the MSE under the UT method and multi-trait models respectively. NRMSE_U and NRMSE_M denote the NRMSE under the UT and multi-trait models respectively. Cor_U and Cor_M denote the Pearson´s correlation under the UT and multi-trait models respectively. RE_MSE, RE_NRMSE and RE_Cor denote the RE computed with the MSE, NRMSE and the Pearson´s correlation, respectively. RE were computed by dividing the prediction performance (with MSE, NRMSE or Cor) of the UT method by the prediction performance of the multi-trait method.

Model	Predictor	DataSet	Env	MSE_U	NRMSE_U	Cor_U	MSE_M	NRMSE_M	Cor_M	RE_MSE	RE_NRMSE	RE_Cor
PLS	E+G+GE	EYT_1	Bed5IR	82.991	2.027	0.567	80.735	2.006	0.594	1.028	1.011	1.048
PLS	E+G+GE	EYT_1	EHT	52.004	1.113	0.498	52.01	1.114	0.531	1	0.999	1.066
PLS	E+G+GE	EYT_1	Flat5IR	11.741	1.086	0.518	11.086	1.064	0.551	1.059	1.021	1.063
PLS	E+G+GE	EYT_1	LHT	758.697	6.833	0.479	756.604	6.83	0.504	1.003	1	1.053
PLS	E+G+GE	EYT_1	Global	226.358	2.765	0.515	225.108	2.753	0.545	1.006	1.004	1.057
PLS	E+G	EYT_1	Bed5IR	77.966	1.978	0.621	76.752	1.97	0.646	1.016	1.004	1.041
PLS	E+G	EYT_1	EHT	46.029	1.072	0.535	44.315	1.05	0.592	1.039	1.022	1.107
PLS	E+G	EYT_1	Flat5IR	14.888	1.211	0.555	14.417	1.196	0.584	1.033	1.012	1.052
PLS	E+G	EYT_1	LHT	767.36	6.879	0.499	767.081	6.875	0.504	1	1.001	1.009
PLS	E+G	EYT_1	Global	226.561	2.785	0.553	225.641	2.773	0.582	1.004	1.004	1.053
PLS	G+GE	EYT_1	Bed5IR	80.518	1.994	0.651	81.427	2.021	0.587	0.989	0.987	0.9
PLS	G+GE	EYT_1	EHT	216.266	2.311	0.558	217.461	2.319	0.553	0.995	0.996	0.991
PLS	G+GE	EYT_1	Flat5IR	66.252	2.158	0.554	67.414	2.186	0.495	0.983	0.987	0.893
PLS	G+GE	EYT_1	LHT	820.858	7.149	0.467	817.73	7.14	0.508	1.004	1.001	1.089
PLS	G+GE	EYT_1	Global	295.973	3.403	0.558	296.008	3.416	0.536	1	0.996	0.961
GBLUP	E+G+GE	EYT_1	Bed5IR	108.19	1.838	0.668	99.977	2.007	0.691	1.082	0.916	1.035
GBLUP	E+G+GE	EYT_1	EHT	70.183	1.248	0.575	85.011	1.643	0.627	0.826	0.76	1.091

GBLUP	E+G+GE	EYT_1	Flat5IR	367.031	4.349	0.591	40.353	2.254	0.593	9.095	1.929	1.003
GBLUP	E+G+GE	EYT_1	LHT	38.394	2.589	0.52	802.585	5.552	0.518	0.048	0.466	0.996
GBLUP	E+G+GE	EYT_1	Global	145.949	2.506	0.588	256.982	2.864	0.607	0.568	0.875	1.032
GBLUP	E+G	EYT_1	Bed5IR	420.228	3.374	0.668	64.187	1.841	0.676	6.547	1.833	1.011
GBLUP	E+G	EYT_1	EHT	448.611	2.923	0.576	73.228	1.45	0.604	6.126	2.016	1.049
GBLUP	E+G	EYT_1	Flat5IR	21.812	2.949	0.591	120.329	2.634	0.591	0.181	1.12	1
GBLUP	E+G	EYT_1	LHT	891.974	5.237	0.518	430.632	4.295	0.515	2.071	1.219	0.995
GBLUP	E+G	EYT_1	Global	445.656	3.621	0.588	172.094	2.555	0.597	2.59	1.417	1.014
GBLUP	G+GE	EYT_1	Bed5IR	85.993	2.053	0.491	71.784	1.892	0.32	1.198	1.085	0.653
GBLUP	G+GE	EYT_1	EHT	222.483	2.343	0.403	201.078	2.232	0.334	1.106	1.05	0.829
GBLUP	G+GE	EYT_1	Flat5IR	66.277	2.164	0.427	79.358	2.48	0.324	0.835	0.872	0.759
GBLUP	G+GE	EYT_1	LHT	820.31	7.148	0.481	821.43	7.158	0.507	0.999	0.999	1.054
GBLUP	G+GE	EYT_1	Global	298.766	3.427	0.45	293.412	3.441	0.371	1.018	0.996	0.824

SUPLEMENTARY TABLE B1

Prediction performance across traits for each environment and across environments (Global) of **dataset 2** (Groundnut) in terms of mean square error (MSE), normalized root mean square error (NRMSE), Person´s correlation (Cor) and relative efficiency (RE) under three predictors (E+G; environments plus genotypes, E+G+GE, that also contain the genotype by environment interaction and G+GE that contain the genotypes and also the genotype by environment interaction). MSE_P and MSE_G denote the MSE under the MT-PLS and MT-GBLUP method. NRMSE_P and NRMSE_G denote the NRMSE under the MT-PLS and MT-GBLUP method. Cor_P and Cor_G denote the Person´s correlation under the MT-PLS and MT-GBLUP method. RE_MSE, RE_NRMSE and RE_Cor denote the RE computed with the MSE, NRMSE and the Person´s correlation respectively. RE were computed by dividing the prediction performance (with MSE, NRMSE or Cor) of the MT-GBLUP method by the prediction performance of the MT-PLS method.

Predictor	DataSet	Env	MSE_P	NRMSE_P	Cor_P	MSE_G	NRMSE_G	Cor_G	RE_MSE	RE_NRMSE	RE_Cor
E+G+GE	Groundnut	ALIYARNAGAR_R15	101937.201	1.032	0.335	96208.041	1.109	0.404	0.944	1.075	0.829
E+G+GE	Groundnut	ICRISAT_PR15-16	141118.182	1.410	0.342	414423.053	2.502	0.356	2.937	1.774	0.961
E+G+GE	Groundnut	ICRISAT_R15	72803.297	0.858	0.565	105199.284	1.699	0.605	1.445	1.980	0.934
E+G+GE	Groundnut	JALGOAN_R15	120712.252	0.915	0.470	113450.369	1.433	0.503	0.940	1.566	0.934
E+G+GE	Groundnut	Global	109142.733	1.054	0.428	182320.187	1.686	0.467	1.670	1.600	0.916
E+G	Groundnut	ALIYARNAGAR_R15	101509.656	1.017	0.375	88502.706	1.251	0.386	0.872	1.230	0.972
E+G	Groundnut	ICRISAT_PR15-16	166719.230	1.494	0.335	155378.714	1.658	0.329	0.932	1.110	1.018
E+G	Groundnut	ICRISAT_R15	76435.515	0.863	0.556	66986.538	1.553	0.619	0.876	1.800	0.898
E+G	Groundnut	JALGOAN_R15	117203.958	0.877	0.519	212365.683	1.249	0.509	1.812	1.424	1.020
E+G	Groundnut	Global	115467.090	1.063	0.446	130808.410	1.428	0.461	1.133	1.343	0.967
G+GE	Groundnut	ALIYARNAGAR_R15	103388.499	1.030	0.335	167094.975	1.088	0.388	1.616	1.056	0.863
G+GE	Groundnut	ICRISAT_PR15-16	141330.327	1.401	0.338	250530.512	1.229	0.340	1.773	0.877	0.994
G+GE	Groundnut	ICRISAT_R15	72674.777	0.867	0.565	113615.278	0.921	0.538	1.563	1.062	1.050
G+GE	Groundnut	JALGOAN_R15	117238.222	0.948	0.489	153501.735	1.174	0.486	1.309	1.238	1.006
G+GE	Groundnut	Global	108657.956	1.061	0.432	171185.625	1.103	0.438	1.575	1.040	0.986

SUPLEMENTARY TABLE B2

Prediction performance across traits for each environment and across environments (Global) of **dataset 2** (Groundnut) in terms of mean square error (MSE), normalized root mean square error (NRMSE), Pearson´s correlation (Cor) and relative efficiency (RE) under three predictors (E+G; environments plus genotypes, E+G+GE, that also contain the genotype by environment interaction and G+GE that contain the genotypes and also the genotype by environment interaction) and two models (MT-PLS and MT-GBLUP). MSE_U and MSE_M denote the MSE under the UT method and multi-trait models respectively. NRMSE_U and NRMSE_M denote the NRMSE under the UT and multi-trait models respectively. Cor_U and Cor_M denote the Pearson´s correlation under the UT and multi-trait models respectively. RE_MSE, RE_NRMSE and RE_Cor denote the RE computed with the MSE, NRMSE and the Pearson´s correlation, respectively. RE were computed by dividing the prediction performance (with MSE, NRMSE or Cor) of the UT method by the prediction performance of the multi-trait method.

Model	Predictor	DataSet	Env	MSE_U	NRMSE_U	Cor_U	MSE_M	NRMSE_M	Cor_M	RE_MSE	RE_NRMSE	RE_Cor
PLS	E+G+GE	Groundnut	ALIYARNAGAR_R15	91429.329	1.04	0.382	101937.201	1.032	0.335	0.897	1.008	0.876
PLS	E+G+GE	Groundnut	ICRISAT_PR15-16	157773.844	1.356	0.287	141118.182	1.41	0.342	1.118	0.962	1.19
PLS	E+G+GE	Groundnut	ICRISAT_R15	72803.074	0.855	0.553	72803.297	0.858	0.565	1	0.997	1.022
PLS	E+G+GE	Groundnut	JALGOAN_R15	116642.07	0.918	0.498	120712.252	0.915	0.47	0.966	1.003	0.944
PLS	E+G+GE	Groundnut	Global	109662.079	1.042	0.43	109142.733	1.054	0.428	1.005	0.989	0.995
PLS	E+G	Groundnut	ALIYARNAGAR_R15	113437.516	1.043	0.356	101509.656	1.017	0.375	1.118	1.025	1.055
PLS	E+G	Groundnut	ICRISAT_PR15-16	179745.808	1.535	0.258	166719.23	1.494	0.335	1.078	1.027	1.299
PLS	E+G	Groundnut	ICRISAT_R15	76435.53	0.86	0.571	76435.515	0.863	0.556	1	0.997	0.974
PLS	E+G	Groundnut	JALGOAN_R15	117204.493	0.92	0.411	117203.958	0.877	0.519	1	1.049	1.262
PLS	E+G	Groundnut	Global	121705.837	1.089	0.399	115467.09	1.063	0.446	1.054	1.025	1.119
PLS	G+GE	Groundnut	ALIYARNAGAR_R15	92989.192	1.042	0.376	103388.499	1.03	0.335	0.899	1.012	0.89
PLS	G+GE	Groundnut	ICRISAT_PR15-16	139589.707	1.411	0.329	141330.327	1.401	0.338	0.988	1.007	1.025
PLS	G+GE	Groundnut	ICRISAT_R15	72294.074	0.865	0.525	72674.777	0.867	0.565	0.995	0.998	1.076
PLS	G+GE	Groundnut	JALGOAN_R15	110216.319	0.921	0.498	117238.222	0.948	0.489	0.94	0.971	0.983
PLS	G+GE	Groundnut	Global	103772.323	1.06	0.432	108657.956	1.061	0.432	0.955	0.998	0.999
GBLUP	E+G+GE	Groundnut	ALIYARNAGAR_R15	8902329.19	5.305	0.382	96208.041	1.109	0.404	92.532	4.784	1.059
GBLUP	E+G+GE	Groundnut	ICRISAT_PR15-16	659513.771	1.756	0.305	414423.053	2.502	0.356	1.591	0.702	1.166
GBLUP	E+G+GE	Groundnut	ICRISAT_R15	64679.478	3.196	0.616	105199.284	1.699	0.605	0.615	1.881	0.983

GBLUP	E+G+GE	Groundnut	JALGOAN_R15	6495158	4.082	0.495	113450.369	1.433	0.503	57.251	2.849	1.016
GBLUP	E+G+GE	Groundnut	Global	4030420.11	3.585	0.449	182320.187	1.686	0.467	22.106	2.127	1.039
GBLUP	E+G	Groundnut	ALIYARNAGAR_R15	297339.592	2.02	0.381	88502.706	1.251	0.386	3.36	1.614	1.012
GBLUP	E+G	Groundnut	ICRISAT_PR15-16	955258.298	6.702	0.306	155378.714	1.658	0.329	6.148	4.043	1.077
GBLUP	E+G	Groundnut	ICRISAT_R15	15814800.3	4.667	0.611	66986.538	1.553	0.619	236.089	3.004	1.013
GBLUP	E+G	Groundnut	JALGOAN_R15	1837643.09	5.722	0.498	212365.683	1.249	0.509	8.653	4.581	1.023
GBLUP	E+G	Groundnut	Global	4726260.32	4.778	0.449	130808.41	1.428	0.461	36.131	3.346	1.026
GBLUP	G+GE	Groundnut	ALIYARNAGAR_R15	95030.618	1.001	0.377	167094.975	1.088	0.388	0.569	0.92	1.028
GBLUP	G+GE	Groundnut	ICRISAT_PR15-16	146881.559	1.421	0.304	250530.512	1.229	0.34	0.586	1.156	1.12
GBLUP	G+GE	Groundnut	ICRISAT_R15	61058.448	0.83	0.624	113615.278	0.921	0.538	0.537	0.901	0.863
GBLUP	G+GE	Groundnut	JALGOAN_R15	110177.073	0.933	0.482	153501.735	1.174	0.486	0.718	0.794	1.007
GBLUP	G+GE	Groundnut	Global	103286.924	1.046	0.447	171185.625	1.103	0.438	0.603	0.948	0.98

SUPLEMENTARY TABLE C1

Prediction performance across traits for each environment and across environments (Global) of **dataset 3** (Disease) in terms of mean square error (MSE), normalized root mean square error (NRMSE), Person´s correlation (Cor) and relative efficiency (RE) under three predictors (E+G; environments plus genotypes, E+G+GE, that also contain the genotype by environment interaction and G+GE that contain the genotypes and also the genotype by environment interaction). MSE_P and MSE_G denote the MSE under the MT-PLS and MT-GBLUP method. NRMSE_P and NRMSE_G denote the NRMSE under the MT-PLS and MT-GBLUP method. Cor_P and Cor_G denote the Person´s correlation under the MT-PLS and MT-GBLUP method. RE_MSE, RE_NRMSE and RE_Cor denote the RE computed with the MSE, NRMSE and the Person´s correlation respectively. RE were computed by dividing the prediction performance (with MSE, NRMSE or Cor) of the MT-GBLUP method by the prediction performance of the MT-PLS method.

Predictor	DataSet	Env	MSE_P	NRMSE_P	Cor_P	MSE_G	NRMSE_G	Cor_G	RE_MSE	RE_NRMSE	RE_Cor
E+G+GE	Disease	Env1	10.314	0.832	0.578	57.023	1.802	0.576	5.529	2.166	1.003
E+G+GE	Disease	Env2	9.647	0.839	0.554	407.110	4.903	0.564	42.201	5.844	0.982
E+G+GE	Disease	Env3	8.697	0.837	0.597	225.761	4.035	0.599	25.958	4.821	0.997
E+G+GE	Disease	Env4	8.492	0.828	0.585	56.330	1.698	0.594	6.633	2.051	0.985
E+G+GE	Disease	Env5	8.762	0.892	0.562	139.431	3.080	0.575	15.913	3.453	0.977
E+G+GE	Disease	Env6	8.624	0.890	0.571	135.931	3.113	0.570	15.762	3.498	1.002
E+G+GE	Disease	Global	9.089	0.853	0.575	170.265	3.105	0.580	18.733	3.640	0.991
E+G	Disease	Env1	10.257	0.831	0.585	32.023	1.358	0.574	3.122	1.634	1.019
E+G	Disease	Env2	9.492	0.836	0.572	84.608	2.303	0.562	8.914	2.755	1.018
E+G	Disease	Env3	8.736	0.837	0.611	177.557	2.722	0.598	20.325	3.252	1.022
E+G	Disease	Env4	8.565	0.833	0.595	288.246	3.631	0.592	33.654	4.359	1.005
E+G	Disease	Env5	8.936	0.901	0.577	219.557	4.053	0.574	24.570	4.498	1.005
E+G	Disease	Env6	8.788	0.898	0.583	133.352	2.880	0.568	15.174	3.207	1.026
E+G	Disease	Global	9.129	0.856	0.587	155.890	2.825	0.578	17.076	3.300	1.016
G+GE	Disease	Env1	10.799	0.851	0.550	10.379	0.834	0.574	0.961	0.980	0.958
G+GE	Disease	Env2	10.028	0.856	0.536	9.452	0.830	0.563	0.943	0.970	0.952
G+GE	Disease	Env3	8.840	0.843	0.560	8.387	0.817	0.598	0.949	0.969	0.936
G+GE	Disease	Env4	8.567	0.827	0.568	8.237	0.809	0.590	0.961	0.978	0.963
G+GE	Disease	Env5	8.222	0.865	0.557	7.849	0.845	0.574	0.955	0.977	0.970

G+GE	Disease	Env6	8.214	0.868	0.557	7.898	0.852	0.570	0.962	0.982	0.977
G+GE	Disease	Global	9.111	0.852	0.555	8.700	0.831	0.578	0.955	0.975	0.960

SUPLEMENTARY TABLE C2

Prediction performance across traits for each environment and across environments (Global) of **dataset 3** (Disease) in terms of mean square error (MSE), normalized root mean square error (NRMSE), Pearson´s correlation (Cor) and relative efficiency (RE) under three predictors (E+G; environments plus genotypes, E+G+GE, that also contain the genotype by environment interaction and G+GE that contain the genotypes and also the genotype by environment interaction) and two models (MT-PLS and MT-GBLUP). MSE_U and MSE_M denote the MSE under the UT method and multi-trait models respectively. NRMSE_U and NRMSE_M denote the NRMSE under the UT and multi-trait models respectively. Cor_U and Cor_M denote the Pearson´s correlation under the UT and multi-trait models respectively. RE_MSE, RE_NRMSE and RE_Cor denote the RE computed with the MSE, NRMSE and the Pearson´s correlation, respectively. RE were computed by dividing the prediction performance (with MSE, NRMSE or Cor) of the UT method by the prediction performance of the multi-trait method.

Model	Predictor	DataSet	Env	MSE_U	NRMSE_U	Cor_U	MSE_M	NRMSE_M	Cor_M	RE_MSE	RE_NRMSE	RE_Cor
PLS	E+G+GE	Disease	Env1	11.414	0.875	0.48	10.314	0.832	0.578	1.107	1.051	1.204
PLS	E+G+GE	Disease	Env2	9.819	0.847	0.54	9.647	0.839	0.554	1.018	1.009	1.026
PLS	E+G+GE	Disease	Env3	10.322	0.896	0.481	8.697	0.837	0.597	1.187	1.071	1.24
PLS	E+G+GE	Disease	Env4	8.757	0.839	0.574	8.492	0.828	0.585	1.031	1.013	1.019
PLS	E+G+GE	Disease	Env5	9.716	0.934	0.49	8.762	0.892	0.562	1.109	1.047	1.147
PLS	E+G+GE	Disease	Env6	8.846	0.901	0.532	8.624	0.89	0.571	1.026	1.013	1.075
PLS	E+G+GE	Disease	Global	9.812	0.882	0.516	9.089	0.853	0.575	1.08	1.034	1.113
PLS	E+G	Disease	Env1	10.538	0.843	0.565	10.257	0.831	0.585	1.027	1.014	1.036
PLS	E+G	Disease	Env2	9.771	0.848	0.55	9.492	0.836	0.572	1.029	1.014	1.039
PLS	E+G	Disease	Env3	9.035	0.853	0.586	8.736	0.837	0.611	1.034	1.02	1.042
PLS	E+G	Disease	Env4	8.719	0.839	0.584	8.565	0.833	0.595	1.018	1.008	1.019
PLS	E+G	Disease	Env5	9.151	0.912	0.559	8.936	0.901	0.577	1.024	1.012	1.033
PLS	E+G	Disease	Env6	8.89	0.904	0.563	8.788	0.898	0.583	1.012	1.006	1.036
PLS	E+G	Disease	Global	9.35	0.866	0.568	9.129	0.856	0.587	1.024	1.012	1.034
PLS	G+GE	Disease	Env1	11.773	0.889	0.446	10.799	0.851	0.55	1.09	1.044	1.234
PLS	G+GE	Disease	Env2	10.672	0.881	0.461	10.028	0.856	0.536	1.064	1.029	1.164
PLS	G+GE	Disease	Env3	9.967	0.875	0.455	8.84	0.843	0.56	1.128	1.039	1.229
PLS	G+GE	Disease	Env4	9.713	0.863	0.446	8.567	0.827	0.568	1.134	1.044	1.274
PLS	G+GE	Disease	Env5	9.063	0.905	0.436	8.222	0.865	0.557	1.102	1.047	1.277

PLS	G+GE	Disease	Env6	8.851	0.901	0.46	8.214	0.868	0.557	1.078	1.038	1.211
PLS	G+GE	Disease	Global	10.007	0.886	0.451	9.111	0.852	0.555	1.098	1.04	1.231
GBLUP	E+G+GE	Disease	Env1	254.002	3.883	0.571	57.023	1.802	0.576	4.454	2.155	1.009
GBLUP	E+G+GE	Disease	Env2	213.486	3.049	0.558	407.11	4.903	0.564	0.524	0.622	1.01
GBLUP	E+G+GE	Disease	Env3	342.048	4.806	0.598	225.761	4.035	0.599	1.515	1.191	1.002
GBLUP	E+G+GE	Disease	Env4	136.367	2.647	0.595	56.33	1.698	0.594	2.421	1.559	0.999
GBLUP	E+G+GE	Disease	Env5	315.87	5.302	0.567	139.431	3.08	0.575	2.265	1.722	1.014
GBLUP	E+G+GE	Disease	Env6	29.462	1.465	0.571	135.931	3.113	0.57	0.217	0.471	0.999
GBLUP	E+G+GE	Disease	Global	215.206	3.525	0.577	170.265	3.105	0.58	1.264	1.135	1.005
GBLUP	E+G	Disease	Env1	267.274	4.268	0.57	32.023	1.358	0.574	8.346	3.142	1.007
GBLUP	E+G	Disease	Env2	130.934	2.789	0.558	84.608	2.303	0.562	1.548	1.211	1.007
GBLUP	E+G	Disease	Env3	232.28	3.728	0.597	177.557	2.722	0.598	1.308	1.369	1
GBLUP	E+G	Disease	Env4	18.898	1.263	0.595	288.246	3.631	0.592	0.066	0.348	0.995
GBLUP	E+G	Disease	Env5	107.309	3.037	0.566	219.557	4.053	0.574	0.489	0.749	1.014
GBLUP	E+G	Disease	Env6	72.397	2.39	0.571	133.352	2.88	0.568	0.543	0.83	0.996
GBLUP	E+G	Disease	Global	138.182	2.912	0.576	155.89	2.825	0.578	0.886	1.031	1.003
GBLUP	G+GE	Disease	Env1	10.466	0.837	0.569	10.379	0.834	0.574	1.008	1.004	1.009
GBLUP	G+GE	Disease	Env2	9.546	0.834	0.556	9.452	0.83	0.563	1.01	1.005	1.011
GBLUP	G+GE	Disease	Env3	8.425	0.818	0.597	8.387	0.817	0.598	1.005	1.002	1.002
GBLUP	G+GE	Disease	Env4	8.178	0.806	0.594	8.237	0.809	0.59	0.993	0.996	0.994
GBLUP	G+GE	Disease	Env5	7.95	0.851	0.566	7.849	0.845	0.574	1.013	1.006	1.015
GBLUP	G+GE	Disease	Env6	7.884	0.851	0.57	7.898	0.852	0.57	0.998	0.999	0.999
GBLUP	G+GE	Disease	Global	8.741	0.833	0.575	8.7	0.831	0.578	1.005	1.002	1.005

SUPLEMENTARY TABLE D1

Prediction performance across traits for each environment and across environments (Global) of **dataset 4** (Indica) in terms of mean square error (MSE), normalized root mean square error (NRMSE), Person´s correlation (Cor) and relative efficiency (RE) under three predictors (E+G; environments plus genotypes, E+G+GE, that also contain the genotype by environment interaction and G+GE that contain the genotypes and also the genotype by environment interaction). MSE_P and MSE_G denote the MSE under the MT-PLS and MT-GBLUP method. NRMSE_P and NRMSE_G denote the NRMSE under the MT-PLS and MT-GBLUP method. Cor_P and Cor_G denote the Person´s correlation under the MT-PLS and MT-GBLUP method. RE_MSE, RE_NRMSE and RE_Cor denote the RE computed with the MSE, NRMSE and the Person´s correlation respectively. RE were computed by dividing the prediction performance (with MSE, NRMSE or Cor) of the MT-GBLUP method by the prediction performance of the MT-PLS method.

Predictor	DataSet	Env	MSE_P	NRMSE_P	Cor_P	MSE_G	NRMSE_G	Cor_G	RE_MSE	RE_NRMSE	RE_Cor
E+G+GE	Indica	2010	212443.082	1.015	0.212	223651.327	1.107	0.461	1.053	1.091	0.460
E+G+GE	Indica	2011	406786.950	1.195	0.266	619613.810	1.355	0.492	1.523	1.134	0.541
E+G+GE	Indica	2012	679532.276	1.121	0.346	540611.167	1.013	0.552	0.796	0.904	0.627
E+G+GE	Indica	Global	432920.769	1.110	0.275	461292.101	1.158	0.502	1.066	1.043	0.548
E+G	Indica	2010	249023.741	1.037	0.217	372266.941	1.635	0.448	1.495	1.577	0.484
E+G	Indica	2011	409235.656	1.206	0.228	520026.462	1.476	0.487	1.271	1.224	0.468
E+G	Indica	2012	666037.436	1.116	0.376	567673.544	1.021	0.560	0.852	0.915	0.671
E+G	Indica	Global	441432.278	1.119	0.274	486655.649	1.377	0.499	1.102	1.231	0.549
G+GE	Indica	2010	213482.391	1.000	0.228	294780.626	1.135	0.110	1.381	1.135	2.073
G+GE	Indica	2011	403745.217	1.193	0.266	487288.886	1.176	0.353	1.207	0.986	0.754
G+GE	Indica	2012	618122.317	1.092	0.344	787453.988	1.075	0.293	1.274	0.984	1.174
G+GE	Indica	Global	411783.308	1.095	0.279	523174.500	1.129	0.252	1.271	1.031	1.107

SUPLEMENTARY TABLE D2

Prediction performance across traits for each environment and across environments (Global) of **dataset 4** (Indica) in terms of mean square error (MSE), normalized root mean square error (NRMSE), Pearson´s correlation (Cor) and relative efficiency (RE) under three predictors (E+G; environments plus genotypes, E+G+GE, that also contain the genotype by environment interaction and G+GE that contain the genotypes and also the genotype by environment interaction) and two models (MT-PLS and MT-GBLUP). MSE_U and MSE_M denote the MSE under the UT method and multi-trait models respectively. NRMSE_U and NRMSE_M denote the NRMSE under the UT and multi-trait models respectively. Cor_U and Cor_M denote the Pearson´s correlation under the UT and multi-trait models respectively. RE_MSE, RE_NRMSE and RE_Cor denote the RE computed with the MSE, NRMSE and the Pearson´s correlation, respectively. RE were computed by dividing the prediction performance (with MSE, NRMSE or Cor) of the UT method by the prediction performance of the multi-trait method.

Model	Predictor	DataSet	Env	MSE_U	NRMSE_U	Cor_U	MSE_M	NRMSE_M	Cor_M	RE_MSE	RE_NRMSE	RE_Cor
PLS	E+G+GE	Indica	2010	212442.661	0.983	0.384	212443.082	1.015	0.212	1	0.969	0.552
PLS	E+G+GE	Indica	2011	418118.199	1.171	0.431	406786.95	1.195	0.266	1.028	0.98	0.618
PLS	E+G+GE	Indica	2012	679530.174	1.064	0.492	679532.276	1.121	0.346	1	0.949	0.703
PLS	E+G+GE	Indica	Global	436697.012	1.073	0.435	432920.769	1.11	0.275	1.009	0.966	0.631
PLS	E+G	Indica	2010	249023.517	1.03	0.358	249023.741	1.037	0.217	1	0.993	0.606
PLS	E+G	Indica	2011	406481.806	1.19	0.427	409235.656	1.206	0.228	0.993	0.987	0.533
PLS	E+G	Indica	2012	666035.176	1.046	0.533	666037.436	1.116	0.376	1	0.937	0.705
PLS	E+G	Indica	Global	440513.499	1.088	0.44	441432.278	1.119	0.274	0.998	0.972	0.622
PLS	G+GE	Indica	2010	216327.792	0.976	0.38	213482.391	1	0.228	1.013	0.975	0.599
PLS	G+GE	Indica	2011	403744.836	1.175	0.384	403745.217	1.193	0.266	1	0.985	0.692
PLS	G+GE	Indica	2012	618120.99	1.05	0.476	618122.317	1.092	0.344	1	0.961	0.721
PLS	G+GE	Indica	Global	412731.206	1.067	0.413	411783.308	1.095	0.279	1.002	0.974	0.675
GBLUP	E+G+GE	Indica	2010	167771.146	0.943	0.466	223651.327	1.107	0.461	0.75	0.852	0.99
GBLUP	E+G+GE	Indica	2011	426226.554	1.151	0.509	619613.81	1.355	0.492	0.688	0.849	0.966
GBLUP	E+G+GE	Indica	2012	693147.711	1.033	0.565	540611.167	1.013	0.552	1.282	1.019	0.976
GBLUP	E+G+GE	Indica	Global	429048.47	1.042	0.513	461292.101	1.158	0.502	0.93	0.9	0.977
GBLUP	E+G	Indica	2010	240391.422	0.993	0.447	372266.941	1.635	0.448	0.646	0.607	1.003
GBLUP	E+G	Indica	2011	390753.698	1.141	0.486	520026.462	1.476	0.487	0.751	0.773	1.003

GBLUP	E+G	Indica	2012	676408.161	1.037	0.561	567673.544	1.021	0.56	1.192	1.016	1
GBLUP	E+G	Indica	Global	435851.094	1.057	0.498	486655.649	1.377	0.499	0.896	0.768	1.002
GBLUP	G+GE	Indica	2010	170971.544	0.967	0.436	294780.626	1.135	0.11	0.58	0.853	0.252
GBLUP	G+GE	Indica	2011	426879.267	1.148	0.481	487288.886	1.176	0.353	0.876	0.976	0.735
GBLUP	G+GE	Indica	2012	662692.029	1.035	0.549	787453.988	1.075	0.293	0.842	0.963	0.534
GBLUP	G+GE	Indica	Global	420180.946	1.05	0.489	523174.5	1.129	0.252	0.803	0.93	0.516

SUPLEMENTARY TABLE E1

Prediction performance across traits for each environment and across environments (Global) of **dataset 5** (Japonica) in terms of mean square error (MSE), normalized root mean square error (NRMSE), Person´s correlation (Cor) and relative efficiency (RE) under three predictors (E+G; environments plus genotypes, E+G+GE, that also contain the genotype by environment interaction and G+GE that contain the genotypes and also the genotype by environment interaction). MSE_P and MSE_G denote the MSE under the MT-PLS and MT-GBLUP method. NRMSE_P and NRMSE_G denote the NRMSE under the MT-PLS and MT-GBLUP method. Cor_P and Cor_G denote the Person´s correlation under the MT-PLS and MT-GBLUP method. RE_MSE, RE_NRMSE and RE_Cor denote the RE computed with the MSE, NRMSE and the Person´s correlation respectively. RE were computed by dividing the prediction performance (with MSE, NRMSE or Cor) of the MT-GBLUP method by the prediction performance of the MT-PLS method.

Predictor	DataSet	Env	MSE_P	NRMSE_P	Cor_P	MSE_G	NRMSE_G	Cor_G	RE_MSE	RE_NRMSE	RE_Cor
E+G+GE	Japonica	2009	198499.628	2.315	0.360	153530.618	2.133	0.468	0.773	0.921	0.769
E+G+GE	Japonica	2010	957145.919	2.987	0.391	647082.593	3.038	0.593	0.676	1.017	0.659
E+G+GE	Japonica	2011	875043.094	1.457	0.283	614513.570	1.309	0.576	0.702	0.898	0.491
E+G+GE	Japonica	2012	336316.702	2.477	0.287	308742.449	2.409	0.491	0.918	0.973	0.585
E+G+GE	Japonica	2013	129759.073	1.032	0.469	96595.936	0.849	0.626	0.744	0.823	0.749
E+G+GE	Japonica	Global	499352.883	2.054	0.358	364093.033	1.948	0.551	0.729	0.948	0.650
E+G	Japonica	2009	182553.916	2.282	0.365	477086.018	2.573	0.524	2.613	1.128	0.697
E+G	Japonica	2010	927889.069	3.026	0.350	873828.710	2.868	0.601	0.942	0.948	0.582
E+G	Japonica	2011	875963.768	1.459	0.287	737267.349	1.351	0.584	0.842	0.926	0.491
E+G	Japonica	2012	339455.998	2.479	0.282	488598.577	2.432	0.493	1.439	0.981	0.572
E+G	Japonica	2013	112702.818	1.003	0.468	118312.500	0.969	0.639	1.050	0.966	0.732
E+G	Japonica	Global	487713.114	2.050	0.350	539018.631	2.039	0.568	1.105	0.995	0.616
G+GE	Japonica	2009	928568.128	2.558	0.185	1463465.636	2.856	0.389	1.576	1.116	0.476
G+GE	Japonica	2010	1097296.155	2.837	0.308	1137874.849	3.103	0.287	1.037	1.094	1.073
G+GE	Japonica	2011	1093856.367	1.739	0.308	1155581.365	1.751	0.369	1.056	1.007	0.835
G+GE	Japonica	2012	388110.145	2.510	0.128	341745.776	2.434	0.442	0.881	0.970	0.290
G+GE	Japonica	2013	402549.435	1.354	0.359	611860.601	1.488	0.460	1.520	1.099	0.780
G+GE	Japonica	Global	782076.046	2.200	0.257	942105.646	2.326	0.389	1.205	1.057	0.661

SUPLEMENTARY TABLE B1

Prediction performance across traits for each environment and across environments (Global) of **dataset 5** (Japonica) in terms of mean square error (MSE), normalized root mean square error (NRMSE), Pearson´s correlation (Cor) and relative efficiency (RE) under three predictors (E+G; environments plus genotypes, E+G+GE, that also contain the genotype by environment interaction and G+GE that contain the genotypes and also the genotype by environment interaction) and two models (MT-PLS and MT-GBLUP). MSE_U and MSE_M denote the MSE under the UT method and multi-trait models respectively. NRMSE_U and NRMSE_M denote the NRMSE under the UT and multi-trait models respectively. Cor_U and Cor_M denote the Pearson´s correlation under the UT and multi-trait models respectively. RE_MSE, RE_NRMSE and RE_Cor denote the RE computed with the MSE, NRMSE and the Pearson´s correlation, respectively. RE were computed by dividing the prediction performance (with MSE, NRMSE or Cor) of the UT method by the prediction performance of the multi-trait method.

Model	Predictor	DataSet	Env	MSE_U	NRMSE_U	Cor_U	MSE_M	NRMSE_M	Cor_M	RE_MSE	RE_NRMSE	RE_Cor
PLS	E+G+GE	Japonica	2009	194004.744	2.17	0.469	198499.628	2.315	0.36	0.977	0.937	0.768
PLS	E+G+GE	Japonica	2010	909997.609	3.015	0.568	957145.919	2.987	0.391	0.951	1.009	0.689
PLS	E+G+GE	Japonica	2011	853792.191	1.366	0.557	875043.094	1.457	0.283	0.976	0.937	0.508
PLS	E+G+GE	Japonica	2012	355265.958	2.442	0.481	336316.702	2.477	0.287	1.056	0.986	0.595
PLS	E+G+GE	Japonica	2013	104920.466	0.906	0.574	129759.073	1.032	0.469	0.809	0.879	0.819
PLS	E+G+GE	Japonica	Global	483596.194	1.98	0.53	499352.883	2.054	0.358	0.968	0.964	0.676
PLS	E+G	Japonica	2009	189533.99	2.177	0.465	182553.916	2.282	0.365	1.038	0.954	0.784
PLS	E+G	Japonica	2010	911693.91	2.952	0.564	927889.069	3.026	0.35	0.983	0.975	0.62
PLS	E+G	Japonica	2011	851298.196	1.373	0.56	875963.768	1.459	0.287	0.972	0.941	0.513
PLS	E+G	Japonica	2012	355668.434	2.445	0.466	339455.998	2.479	0.282	1.048	0.986	0.606
PLS	E+G	Japonica	2013	97466.269	0.958	0.59	112702.818	1.003	0.468	0.865	0.954	0.794
PLS	E+G	Japonica	Global	481132.16	1.981	0.529	487713.114	2.05	0.35	0.987	0.966	0.663
PLS	G+GE	Japonica	2009	928563.87	2.499	0.416	928568.128	2.558	0.185	1	0.977	0.445
PLS	G+GE	Japonica	2010	1097294.72	2.814	0.38	1097296.16	2.837	0.308	1	0.992	0.809
PLS	G+GE	Japonica	2011	1034427.43	1.685	0.377	1093856.37	1.739	0.308	0.946	0.969	0.817
PLS	G+GE	Japonica	2012	396290.951	2.483	0.421	388110.145	2.51	0.128	1.021	0.989	0.304
PLS	G+GE	Japonica	2013	402545.528	1.318	0.489	402549.435	1.354	0.359	1	0.973	0.735
PLS	G+GE	Japonica	Global	771824.5	2.16	0.416	782076.046	2.2	0.257	0.987	0.982	0.618
GBLUP	E+G+GE	Japonica	2009	156744.435	1.893	0.483	153530.618	2.133	0.468	1.021	0.887	0.969

GBLUP	E+G+GE	Japonica	2010	931163.193	3.169	0.593	647082.593	3.038	0.593	1.439	1.043	1
GBLUP	E+G+GE	Japonica	2011	967668.657	1.46	0.601	614513.57	1.309	0.576	1.575	1.116	0.959
GBLUP	E+G+GE	Japonica	2012	406587.357	2.414	0.534	308742.449	2.409	0.491	1.317	1.002	0.92
GBLUP	E+G+GE	Japonica	2013	95950.279	1.007	0.627	96595.936	0.849	0.626	0.993	1.186	0.998
GBLUP	E+G+GE	Japonica	Global	511622.784	1.989	0.568	364093.033	1.948	0.551	1.405	1.021	0.97
GBLUP	E+G	Japonica	2009	224437.585	2.492	0.544	477086.018	2.573	0.524	0.47	0.968	0.963
GBLUP	E+G	Japonica	2010	922103.901	4.357	0.594	873828.71	2.868	0.601	1.055	1.519	1.011
GBLUP	E+G	Japonica	2011	732041.101	1.366	0.598	737267.349	1.351	0.584	0.993	1.011	0.977
GBLUP	E+G	Japonica	2012	243663.367	2.409	0.521	488598.577	2.432	0.493	0.499	0.991	0.945
GBLUP	E+G	Japonica	2013	88406.968	1.265	0.639	118312.5	0.969	0.639	0.747	1.305	1.001
GBLUP	E+G	Japonica	Global	442130.584	2.378	0.579	539018.631	2.039	0.568	0.82	1.166	0.981
GBLUP	G+GE	Japonica	2009	1219258.69	2.737	0.379	1463465.64	2.856	0.389	0.833	0.958	1.025
GBLUP	G+GE	Japonica	2010	1077903.02	2.871	0.465	1137874.85	3.103	0.287	0.947	0.925	0.617
GBLUP	G+GE	Japonica	2011	1147158.94	1.729	0.411	1155581.37	1.751	0.369	0.993	0.987	0.897
GBLUP	G+GE	Japonica	2012	343441.355	2.446	0.486	341745.776	2.434	0.442	1.005	1.005	0.909
GBLUP	G+GE	Japonica	2013	446628.063	1.359	0.453	611860.601	1.488	0.46	0.73	0.913	1.017
GBLUP	G+GE	Japonica	Global	846878.012	2.228	0.439	942105.646	2.326	0.389	0.899	0.958	0.887
